# The fine-scale genetic structure and evolution of the Japanese population

**DOI:** 10.1371/journal.pone.0185487

**Published:** 2017-11-01

**Authors:** Fumihiko Takeuchi, Tomohiro Katsuya, Ryosuke Kimura, Toru Nabika, Minoru Isomura, Takayoshi Ohkubo, Yasuharu Tabara, Ken Yamamoto, Mitsuhiro Yokota, Xuanyao Liu, Woei-Yuh Saw, Dolikun Mamatyusupu, Wenjun Yang, Shuhua Xu, Japanese Genome Variation Consortium, Yik-Ying Teo, Norihiro Kato

**Affiliations:** 1 Department of Gene Diagnostics and Therapeutics, National Center for Global Health and Medicine, Tokyo, Japan; 2 Department of Clinical Gene Therapy, Osaka University Graduate School of Medicine, Suita, Japan; 3 Department of Human Biology and Anatomy, Graduate School of Medicine, University of the Ryukyus, Nishihara-cho, Japan; 4 Department of Functional Pathology, Shimane University School of Medicine, Izumo, Japan; 5 Department of Hygiene and Public Health, Teikyo University School of Medicine, Tokyo, Japan; 6 Center for Genomic Medicine, Kyoto University Graduate School of Medicine, Kyoto, Japan; 7 Department of Medical Chemistry, Kurume University School of Medicine, Kurume, Japan; 8 Department of Genome Science, School of Dentistry, Aichi Gakuin University, Nagoya, Japan; 9 Saw Swee Hock School of Public Health, National University of Singapore, Singapore; 10 NUS Graduate School for Integrative Science and Engineering, National University of Singapore, Singapore, Singapore; 11 Life Sciences Institute, National University of Singapore, Singapore, Singapore; 12 College of the Life Sciences and Technology, Xinjiang University, Urumqi, China; 13 Key Laboratory of Reproduction and Heredity of Ningxia Region, Ningxia Medical University, Yinchuan, Ningxia, China; 14 Max Planck Independent Research Group on Population Genomics, Chinese Academy of Sciences and Max Planck Society Partner Institute for Computational Biology, Shanghai Institutes for Biological Sciences, Chinese Academy of Sciences Shanghai, China; 15 School of Life Sciences and Technology, ShanghaiTech University, Shanghai, China; 16 Collaborative Innovation Center of Genetics and Development, Shanghai, China; 17 Genome Institute of Singapore, Agency for Science, Technology and Research, Singapore, Singapore; 18 Department of Statistics and Applied Probability, National University of Singapore, Singapore, Singapore; Universitat Pompeu Fabra, SPAIN

## Abstract

The contemporary Japanese populations largely consist of three genetically distinct groups—Hondo, Ryukyu and Ainu. By principal-component analysis, while the three groups can be clearly separated, the Hondo people, comprising 99% of the Japanese, form one almost indistinguishable cluster. To understand fine-scale genetic structure, we applied powerful haplotype-based statistical methods to genome-wide single nucleotide polymorphism data from 1600 Japanese individuals, sampled from eight distinct regions in Japan. We then combined the Japanese data with 26 other Asian populations data to analyze the shared ancestry and genetic differentiation. We found that the Japanese could be separated into nine genetic clusters in our dataset, showing a marked concordance with geography; and that major components of ancestry profile of Japanese were from the Korean and Han Chinese clusters. We also detected and dated admixture in the Japanese. While genetic differentiation between Ryukyu and Hondo was suggested to be caused in part by positive selection, genetic differentiation among the Hondo clusters appeared to result principally from genetic drift. Notably, in Asians, we found the possibility that positive selection accentuated genetic differentiation among distant populations but attenuated genetic differentiation among close populations. These findings are significant for studies of human evolution and medical genetics.

## Introduction

The contemporary Japanese populations largely consist of three genetically distinct groups—Hondo, Ryukyu and Ainu [1]. The Hondo people, comprising 99% of the Japanese, live in the four main islands of Japan; the Ryukyu people, comprising 1%, live in Okinawa, the southwestern archipelago; and the Ainu people are indigenous to the northernmost islands of Japan and currently comprise ~0.03% of the Japanese. It has been shown that the three groups are clearly separated by principal-component analysis (PCA) using genome-wide single nucleotide polymorphism (SNP) genotypes [2,3], where the Hondo people form one large, almost indistinguishable cluster, albeit being sampled from various locations of Japan.

The currently accepted model for the history of Japanese populations is the ‘dual structure model’ [1,4–6]. This model assumes that there were two waves of human migrations from the Korean Peninsula or the Asian continent to the Japanese Archipelago. The latter could be divided into more than one wave, as some authors [6] addressed in the three migration waves model. The first wave of migrations by hunter-gatherers took place from 40,000 Before Present (BP); the origin of the early migrants (i.e., Jomon people) is assumed to be Southeast Asia in the dual structure model but remains to be determined. In the second wave of migrations from 3,000 BP, farmers migrated from the Korean Peninsula to Kyushu, southernmost of the four main islands of Japan, and became the later migrants (i.e., Yayoi people). Then, the Yayoi people gradually spread and admixed with the Jomon people. Supporting the model, genetic similarity to Korean people was found to be higher in the Hondo people than in the Ryukyu and Ainu people (who presumably retain more Jomon ancestry components) in DNA studies of mitochondria [7,8], Y chromosome [9–11] and classic genetic markers [12] in modern people. Ancient mitochondrial DNA analysis of Jomon and Yayoi people also agree with the model [13,14]. Based on genome-wide SNP analysis of modern Japanese, the proportion of Jomon ancestry in the modern Japanese was estimated to be approximately 18% or 28% for the Hondo and Ryukyu people, respectively [15], and higher for the Ainu people [16]. Such a gradual spread of the Yayoi people may result in some genetic heterogeneity among the Hondo people. Looking closer, Hondo people have been observed to be heterogeneous in body shape and in the transition from foraging to agriculture.

As an increasing amount of genetic data have become available during the past decade, a variety of analytical methods have been developed to detect population structure; e.g., modeling of ancestral allele frequency [17] and the PCA of genotype similarity [18], using unlinked DNA markers. Among those developed to date, the fineSTRUCTURE method is noted for its capacity that it computes haplotype sharing among the individuals and infers finer-scale population structure by using dense genome-wide SNP data [19]. In fact, the results for the fineSTRUCTURE clustering of the British populations have clearly agreed with geographic regions in the country and have elucidated its European continental origin [20].

Understanding fine-scale genetic structure of Japanese populations is important in the studies of genetic epidemiology and medical genetics, in particular, to control the confounding influences of population stratification on genetic association studies [21]. Therefore, we applied the fineSTRUCTURE method to genome-wide SNP data for 1600 individuals—1400 Hondo people, and 200 Ryukyu people—who were sampled from eight distinct regions of Japan, to investigate their genetic structure (S1 Table). We also combined the Japanese data with 26 other Asian populations data to analyze the shared ancestry. Moreover, we examined what types of evolutionary forces could have shaped the regional genetic differentiation in Japan and other parts of Asia.

## Results

### Genetic structure of Japanese populations

**Fig 1:**
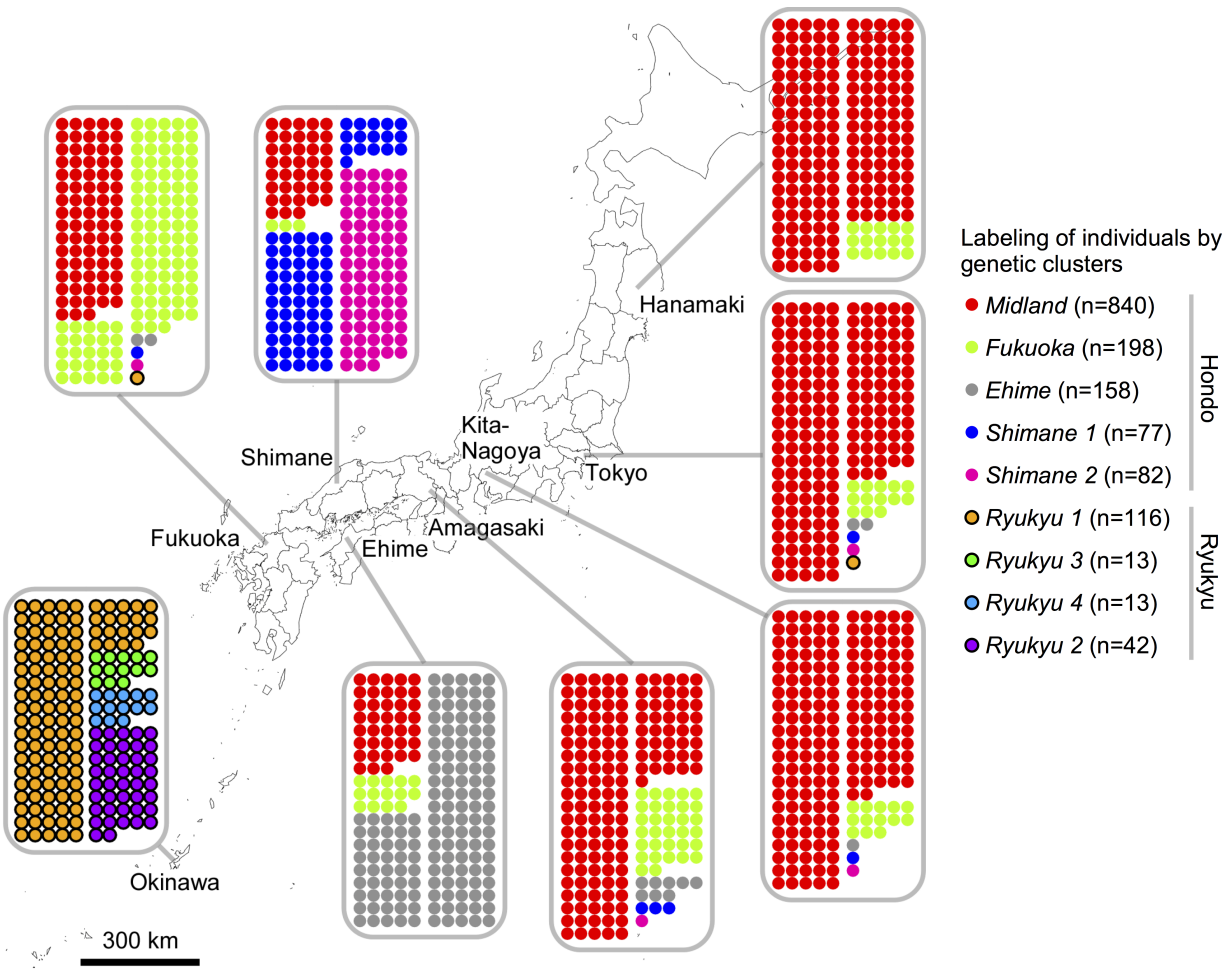
Geographic distribution of the Japanese genetic clusters. Each population is mapped to its geographic location. The individuals in a population are represented by circles colored according to their assigned genetic clusters. The figure map was generated using the packages maps v3.1.0 and mapdata v2.2–6 in the R software.

We successfully grouped 1600 Japanese individuals, who had been enrolled 200 each at eight distinct locations across Japan (*dataset A*), by using only genetic data with the fineSTRUCTURE program. Nine genetic clusters with ≥10 individuals were identified, constituting 1539 (96%) of the Japanese participants. The smaller clusters (<9 individuals) were discarded from further analyses, since they are distinct in the distribution of cluster size. The genetic clusters remarkably well corresponded with geographic regions in Japan (Fig 1). Accordingly, we named the Japanese genetic clusters after the corresponding geographic regions and italicized the cluster names to distinguish them from geographic names; multiple clusters corresponding to one region were then numbered consecutively. Individuals from Okinawa, namely the Ryukyu people, were exclusively assigned to four clusters, which we designated *Ryukyu 1*, *2*, *3* and *4*. The other five clusters consisted of individuals from the main islands of Japan, namely the Hondo people. The *Shimane 1* and *2* clusters almost exclusively consisted of individuals from Shimane prefecture, and the *Ehime* cluster largely consisted of individuals from Ehime prefecture. On the other hand, the remaining two clusters in the Hondo people, *Midland* and *Fukuoka*, consisted of individuals from across the main islands of Japan; a major part of individuals in the middle to northern regions of Japan were assigned to the *Midland* cluster, while those assigned to the *Fukuoka* cluster were most prominent in Fukuoka prefecture. The *Fukuoka* cluster appeared to include a number of individuals who could have been assigned to the *Midland* cluster by chance, although the remaining seven clusters were fairly separated by fineSTRUCTURE (S2 Table).

In previous studies, the two large groups—the Ryukyu and Hondo people—were clearly separated, whereas the Hondo people from different regions of Japan could not be separated further [2], contrary to the finding in the present study. To investigate whether our success in finer clustering that could distinguish geographic regions in the main islands of Japan was due principally to differences in the analyzed sample or methodology, we reanalyzed the dataset by using the EIGENSOFT PCA method [18]. Almost identical to the previous study results, we found that genetic clusters for the Hondo people overlapped each other with slight, though indistinguishable, shift in the EIGENSOFT plot (S1 Fig), indicating that fineSTRUCTURE could outperform EIGENSOFT PCA in resolution. By classifying the individuals in the current study using the principal components for the previous BioBank Japan study [2,22], we confirmed that the primary genetic data agreed well between the two studies (S2 Fig).

**Fig 2:**
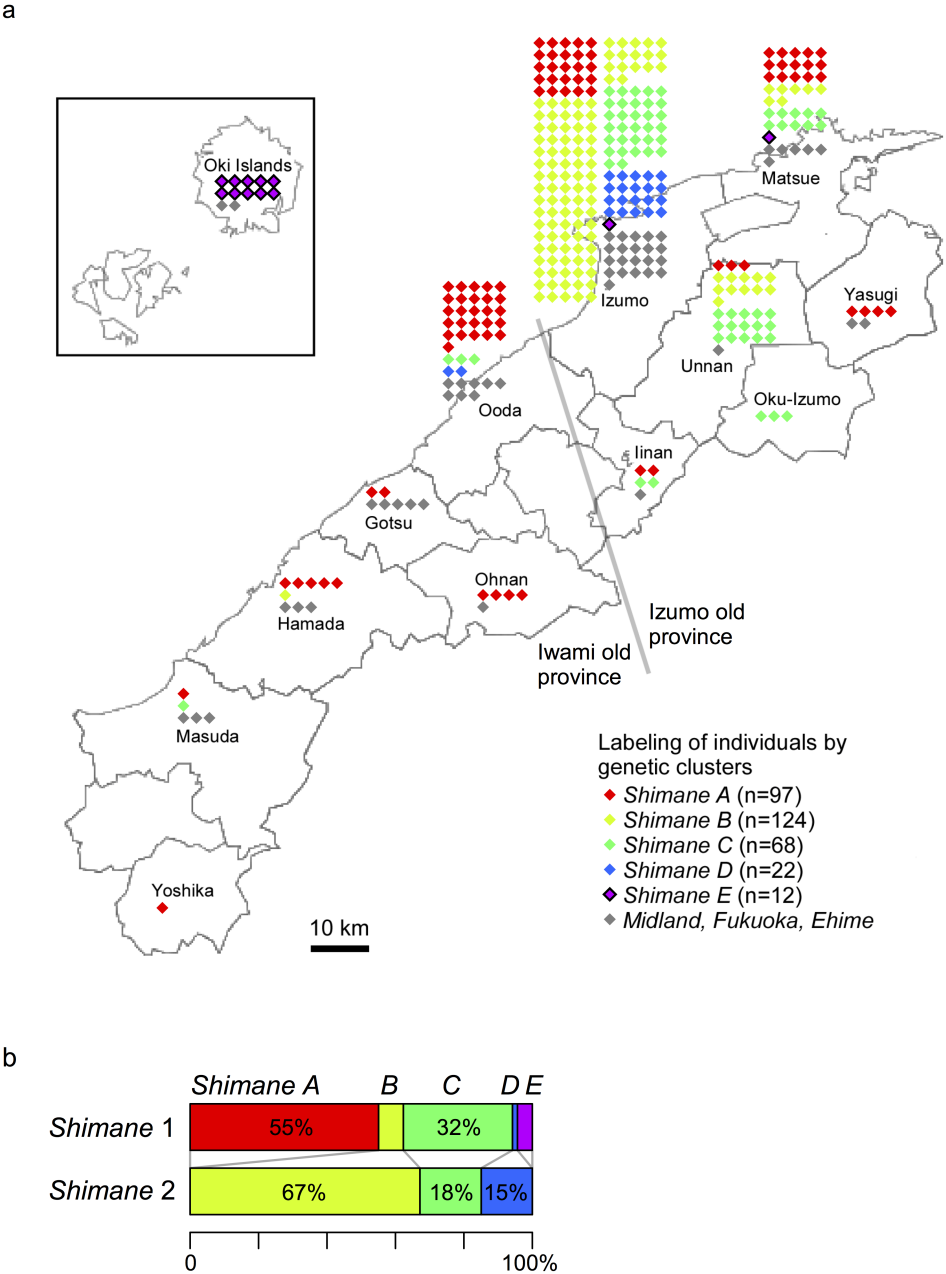
Genetic clustering of an extended set of individuals from the Shimane prefecture. **a**) For each municipality, the individuals therein are represented by diamonds colored according to their assigned genetic clusters. **b**) Re-classification of the individuals in the *Shimane 1*, *2* clusters of Fig 1 into the finer clusters *Shimane A*–*E*. The map of Shimane prefecture (https://commons.wikimedia.org/wiki/File%3AMap_of_Shimane_Prefecture_Ja.svg) is licensed under the Attribution-ShareAlike 3.0 Unported license. The license terms can be found on the following link: https://creativecommons.org/licenses/by-sa/3.0/.

We examined the possibility of identifying more subtle structure of the Hondo people than initially detectable in the modest scale (200 individuals in each region) of *dataset A*. For individuals from Shimane prefecture, who were grouped into multiple clusters in *dataset A* (Fig 1), we reanalyzed *dataset B* that increased the number of individuals, who live in the prefecture, from 200 to 428. The five clusters with ≥10 individuals in *dataset B*, specific to Shimane prefecture, were designated *Shimane A*, *B*, *C*, *D* and *E* (Fig 2a), some of which were found only in restricted parts of the prefecture. For example, all but two individuals in the *Shimane E* cluster were located in the Oki Islands. While the *Shimane A* cluster was distributed across the prefecture, the *Shimane B*, *C* and *D* clusters appeared to be mostly restricted to the north-east part, which corresponds to the Izumo old province, one of the two old provinces that existed in Shimane prefecture >400 years BP. Moreover, the composition of clusters substantially differed between the Izumo and Oota cities, which are only 30 km apart. When increasing the number of individuals in Shimane prefecture, the *Shimane 1* and *2* clusters in *dataset A* were largely subdivided into the *Shimane A*, *C* and *E* clusters and the *Shimane B*, *C* and *D* clusters in *dataset B*, respectively (Fig 2b), allowing for finer genetic clustering with fineSTRUCTURE. Here, it should be also noted that while the *Shimane A* and *E* clusters are considered as sub-cluster of *Shimane 1*, the *Shimane B-D* clusters coexist in both *Shimane 1* and *2* clusters, with the *Shimane C* cluster and the *Shimane B* and *D* clusters being more prominent in the *Shimane 1* and *2* clusters, respectively (Fig 2b). This indicates that even though fineSTRUCTURE may infer finer-scale population structure than EIGENSOFT PCA and allows for distinguishing geographic regions in the main islands of Japan (Fig 1), some part of the clustering is not discretely separable within a given contiguous area.

### Impact of genetic structure on genome-wide association studies (GWAS)

As population stratification can cause spurious signals in GWAS [21], we evaluated the potential impact of genetic differentiation in the Japanese on GWAS. In the simulations using pairs of subpopulations from the Hondo clusters, the values of *λ*_GC_, a genome-wide inflation factor for genomic control [23], which is an indicator of the inflation of false-positive rates due to population stratification, were moderate, i.e., in the range between 1.05 and 1.15 (S3 Table). On the other hand, the *λ*_GC_ values for the pairs between the Ryukyu people (*Ryukyu 1–4* clusters) and any of the Hondo clusters exceeded 2.70, which is beyond a generally acceptable level (*λ*_GC_ ≤ 1.10). Thus, we found that population stratification was strong enough to bias GWAS even within the Hondo people and more seriously between the Hondo and Ryukyu people.

### Ancestry profile and genetic admixture of Japanese populations

To understand the ancestry of the Japanese population, we performed fineSTRUCTURE analysis for 3928 individuals from 34 Asian populations, including the Japanese (*dataset C*). The non-Japanese Asians, which we call continental Asians, formed 23 clusters with ≥8 individuals (S4 Table). The continental Asian clusters are named by a prefix C followed by an arbitrary number, such as C282, which is a cluster formed by Koreans. We measured the genetic differentiation between the clusters by haplotypic F_ST_ and inferred the phylogenetic tree based on the HF_ST_ distance (S3 Fig, S5 Table). The relation between the clusters agreed with the relation between Asian populations reported in previous studies [24,25].

We next inferred the ‘ancestry profile’ for each Japanese cluster by using a coancestry matrix in which donors are limited to continental Asians. The coancestry matrix represents haplotype sharing between each individual, called recipient, and a set of donor individuals. The recipient’s genome is ‘painted’ as a patchwork of genomes from donors who had the most similar sequence to the recipient in local chromosomal regions. The (*i*, *j*) element of the coancestry matrix represents the amount of shared (i.e., painted) haplotypes between recipient *i* and donor *j*. Within this coancestry matrix, we aimed to represent the patterns as to how the components of recipients taken from continental Asian clusters could mix to approximate those taken from a Japanese cluster. By calculating a mixture ratio of the continental Asian clusters formed by modern individuals, we indirectly estimated the proportion of ancestral DNA that was shared with the continental Asian clusters. Since geographic distribution could have differed between modern-day populations (or clusters) and their ancestors, the estimate of ancestry profile cannot provide the definitive history of original migration, unless it will be further verified against historical evidence.

**Fig 3:**
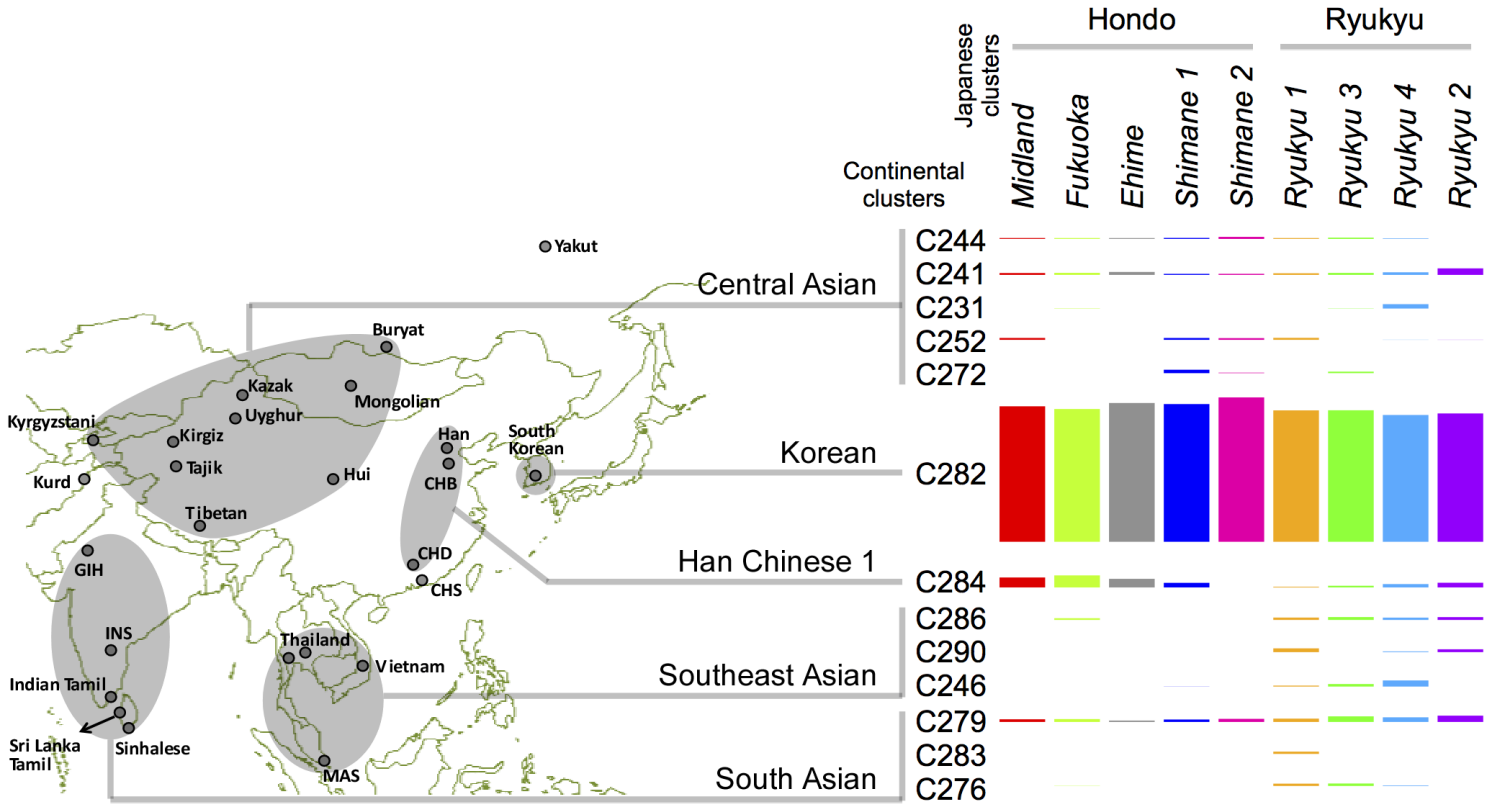
Ancestry profile of the Japanese genetic clusters. For each Japanese cluster, the non-Japanese genetic components are represented as a mixture of continental Asian clusters. The bar graph indicates the proportion of continental Asian clusters contributing to a given Japanese cluster. Continental Asian clusters with ≥1% contribution to at least one Japanese cluster are shown. The map indicates geographic locations of the populations included in continental Asian clusters. See S4 Table for details. The figure map was generated using the packages maps v2.3–9 and mapdata v2.2–3 in the R software.

For any Japanese cluster of the Hondo people, major components of ancestry profile were from the Korean (C282; 87–94%), followed by Han Chinese 1 (C284; 0–8%) (Fig 3, S4 Table), where the Han Chinese 1 cluster was distributed predominantly in northern China. Among the Hondo people, the *Shimane 2* cluster had a larger component from the Korean (94% vs 87–91%) and a smaller component from the Han Chinese 1 (0% vs 3–8%) than other Hondo clusters (*P* < 0.001 comparing *Shimane 2* and *Midland*). In the Ryukyu clusters, on the other hand, as compared to ancestry profile in the Hondo clusters, the components from the Southeast Asian (4–6% vs. 0–1%) and South Asian (4–6% vs. 1–2%) clusters were larger (*P* < 0.001 comparing *Ryukyu 1* and *Midland*), and those from the Korean cluster (83–86% vs. 87–94%), the Han Chinese 1 cluster (0–3% vs. 0–8%) and the Central Asian (3–5% vs. 2–5%) clusters were similar. For the Japanese as a whole, there appeared to be some genetic components from all of the Central, East, Southeast and South Asia.

Next, we investigated the possibility and timing of admixture in forming the Japanese. The ALDER program [26] tests and dates the admixture by measuring admixture-induced linkage disequilibrium in the admixed population based on the following theory. Admixture-induced linkage disequilibrium occurs between two SNPs having different allele frequencies in the admixture ‘source’ populations, and gradually weakens by recombination as generations pass after the initial admixture event. By applying a decay curve of admixture-induced linkage disequilibrium to the data, the possibility of admixture occurrence can be estimated by the statistical significance of fitness of the curve, and the timing of admixture can be estimated by steepness of the curve. In the ALDER program, a pair of ‘reference’ populations are incorporated one at a time such that the reference populations should approximate the two admixture ‘source’ populations (with regards to SNP allele frequency) while they do not have to be direct descendants of the sources. Here, an admixed population itself can be used as one reference of the pair; in this case, the other reference is assumed to be more similar to one source than the other source, thereby allowing for differentiation between the two admixture source populations in question. We thus analyzed a series of combinations between a Japanese cluster and a continental Asian cluster and estimated the admixture time. Statistically significant admixture was detected for reference populations that involved pairs between any of the Japanese clusters and four continental Asian clusters—Korean (C282), Han Chinese (C284 and C291) and Vietnamese (C280) (Table 1; all *P* < 0.05). When either of the continental Asian clusters was used as a reference, the admixture time did not differ significantly (*P* > 0.05 for test of heterogeneity) among five Hondo clusters or among four Ryukyu clusters. However, we found that the admixture timings were significantly heterogeneous (*P* < 0.05) when all Japanese clusters were combined, indicating that admixture events in Hondo and Ryukyu occurred at different timings (S4 Fig). The most accurate estimate (showing the smallest SE) of the admixture time for the Hondo clusters was 49.9 generations ago for the references, *Midland* and C284, and that for the Ryukyu clusters was 39.3 generations ago for the references, *Ryukyu 1* and C291.

**Table 1:** Admixture events detected in the Japanese clusters.

Admixed cluster	Surrogate group approximating one admixture source							
	C282 (Korean)		C284 (Han Chinese 1)		C291 (Han Chinese 2)		C280 (Vietnamese)	
	Admixture time	*P*-value	Admixture time	*P*-value	Admixture time	*P*-value	Admixture time	*P*-value
*Midland*	52.0 ± 3.6	4.3E-17	49.9 ± 3.2	7.1E-36	53.5 ± 3.4	6.1E-25	56.9 ± 7.2	8.4E-10
*Fukuoka*	46.5 ± 3.9	2.0E-10	51.6 ± 5.4	2.9E-14	50.6 ± 6.8	1.4E-12	55.1 ± 10.6	3.0E-05
*Ehime*	50.5 ± 10.0	7.3E-04	37.6 ± 5.0	4.1E-13	47.8 ± 7.2	2.9E-10	42.8 ± 7.4	2.1E-03
*Shimane 1*	58.9 ± 13.1	3.3E-03	51.5 ± 8.4	4.9E-02	58.0 ± 8.8	5.6E-10	51.5 ± 8.4	9.9E-09
*Shimane 2*	33.8 ± 8.3	1.9E-03	46.8 ± 13.3	7.2E-03	44.7 ± 11.0	5.9E-04	40.9 ± 8.8	4.2E-05
*Ryukyu 1*	38.1 ± 2.2	7.2E-49	38.2 ± 2.2	3.0E-64	39.3 ± 1.9	1.9E-60	43.8 ± 2.3	1.0E-69
*Ryukyu 2*	43.1 ± 2.5	2.0E-36	43.5 ± 2.6	1.7E-50	41.4 ± 3.1	7.1E-39	41.5 ± 4.4	1.7E-20
*Ryukyu 3*	47.1 ± 9.2	4.1E-06	54.4 ± 9.4	9.5E-08	53.1 ± 8.1	8.4E-10	55.4 ± 13.3	3.5E-04
*Ryukyu 4*	43.2 ± 8.2	3.3E-05	54.0 ± 10.8	6.2E-06	54.8 ± 10.7	3.3E-06	56.0 ± 12.3	6.8E-05

Admixture time is shown in generations before present (estimate ± SE).

P-values are corrected for multiple testing by continental Asian reference clusters.

**Fig 4:**
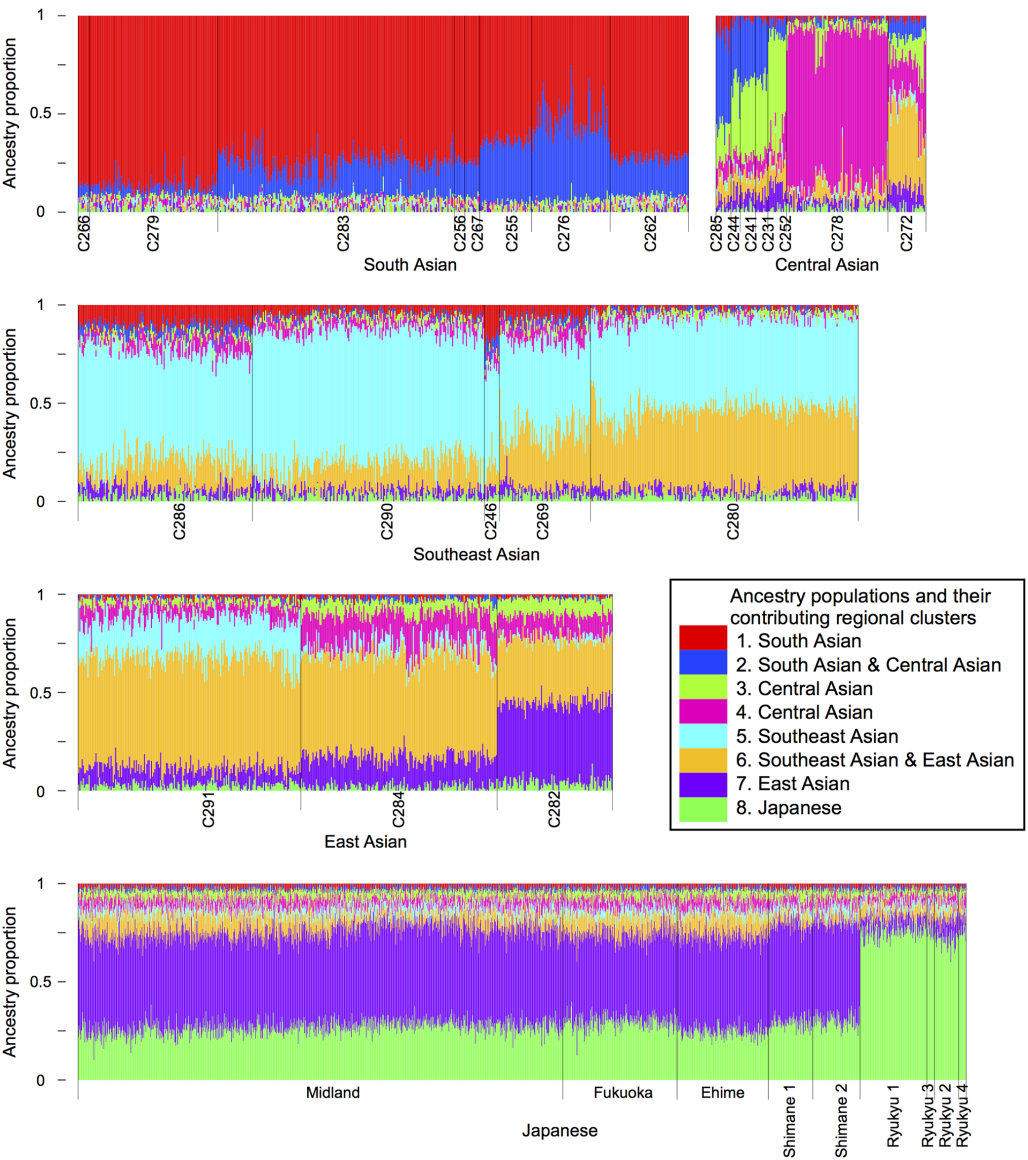
Ancestry populations for the Japanese and continental Asians modeled using the ADMIXTURE program. Eight ancestry populations are inferred. In this bar plot, each individual is represented by a vertical bar, which is colored by eight segments that indicate the proportion contributed by the corresponding ancestry populations. The individuals are sorted by 23 continental Asian clusters (see S4 Table for details) and nine Japanese clusters. The bar widths are halved for Japanese individuals, to fit the plot in space. The inset shows color-codes for eight ancestry populations and geographic areas of clusters, to which the individual population mainly contributes.

To directly model the ancestral populations of Japanese and continental Asians, we performed unsupervised genetic clustering using the ADMIXTURE program [27]. For 3928 Asian individuals including the Japanese (*dataset C*), the inferred model was comprised of eight ancestral populations (Fig 4). Two ancestral populations (6th in gold and 7th in purple, Fig 4) contributed to all East Asians with some regional diversity; their contributing fractions were approximately 8% and 49% to the Hondo people and 5% and 8% to the Ryukyu people, respectively. Another ancestral population (8th in darker green, Fig 4), which was almost specific to the Japanese, contributed 27% to the Hondo people and 75% to the Ryukyu people. Thus, there are marked differences in the contribution of ancestral populations between the Hondo and Ryukyu people, further supporting admixture in the Japanese.

### Positive selection and genetic differentiation

We investigated an issue as to how genetic differentiation between clusters was formed by natural selection and random genetic drift. Beforehand, we examined whether genetic differentiation in the Japanese population was attributable to specific loci (such as HLA on chromosome 6). We computed the coancestry matrix for SNPs on single chromosomes separately and for those across 22 autosomes together in *dataset A* and found a consistent pattern of coancestry matrix that distinguished nine Japanese clusters (S5 Fig). The coancestry matrices for single chromosomes were all positively correlated, and the correlation coefficients were larger between pairs of longer chromosomes (S6 Table), indicating that genetic differentiation in the Japanese was evenly detectable across the genome.

Subsequently, we examined the relationship between genetic differentiation and positive selection. For this, we partitioned the genome into 4-SNPs windows and tested whether some windows showing prominent locus-specific genetic differentiation corresponded to those showing positive selection signature. Specifically, for a defined set of populations, the extent of locus-specific genetic differentiation was measured with HF_ST_ in each 4-SNPs window. According to the HF_ST_ value, the 4-SNPs windows from the genome were sorted into 20 equally-sized bins, such that each bin had the same number of 4-SNPs windows. Chromosomal regions undergoing positive selection were detected by using the HaploPS program [28], which could identify haplotypes showing excess frequency in proportion to their length in each individual population. We did not intend to detect positive selection extensively, but to detect without using the information of genetic differentiation. The HaploPS program was suited for this purpose. Finally, for each bin, we computed the proportion of 4-SNPs windows residing in positively selected regions.

**Fig 5:**
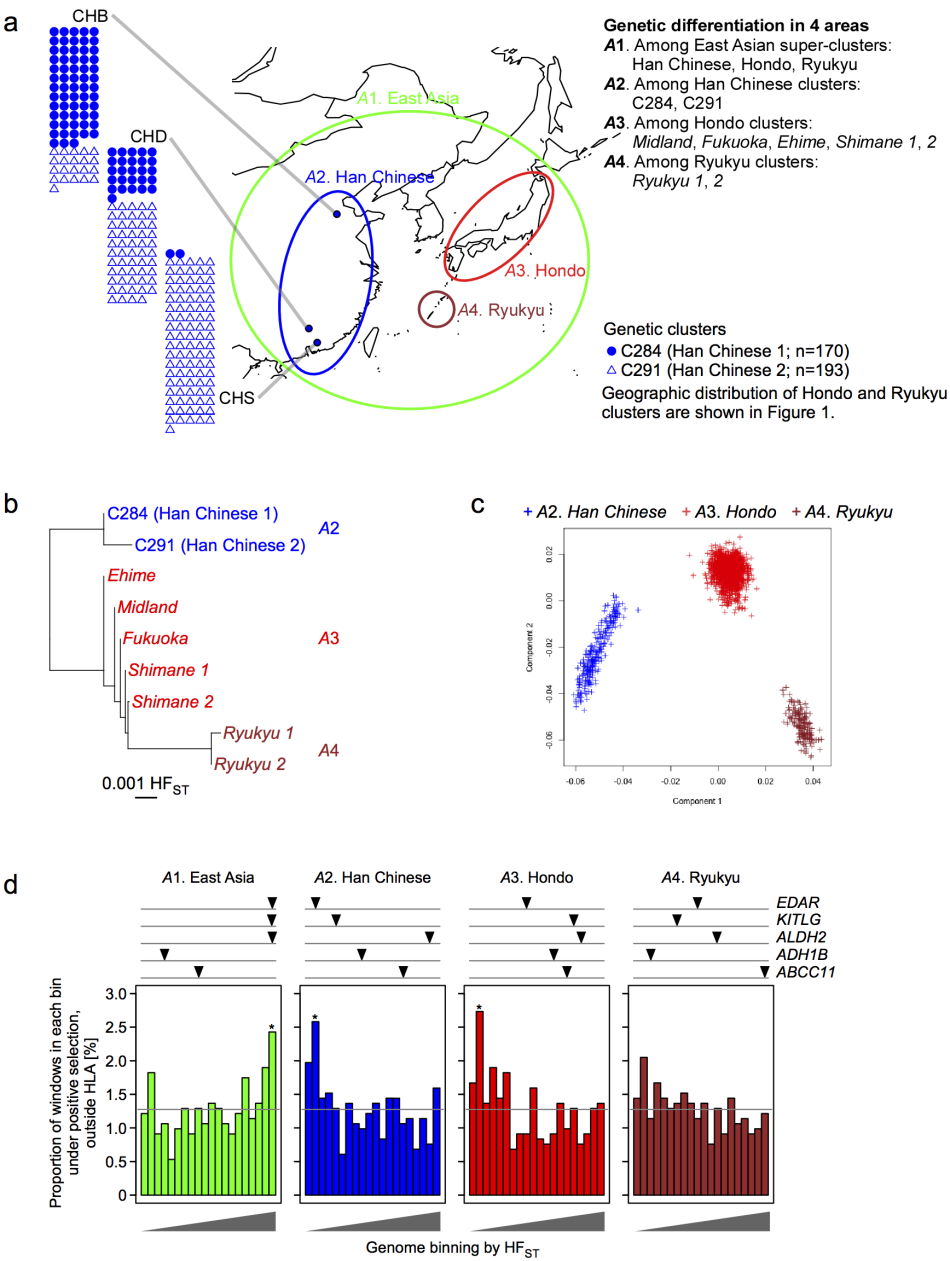
Relation between locus-specific genetic differentiation and positive selection in East Asia. **a**) Genetic differentiation is analyzed in four areas, consisting of different sets of populations. **b**) Neighbor-joining phylogenetic tree of the genetic clusters. **c**) Genetic similarity among the individuals is plotted by the first two principal components of EIGENSOFT PCA. **d**) For each area, 4-SNP windows of the genome are sorted into 20 equal-sized bins arranged according to locus-specific HF_ST_ in the corresponding populations. The proportion of positively selected windows (in vertical axis) is plotted across 20 HF_ST_ bins (in horizontal axis); the average proportion (1.3%) is indicated by a gray line. Asterisk (*) indicates significant enrichment of positively selected windows in a specific bin, compared to the whole-genome average (Fisher’s exact test *P* < 0.01/20). The highest (or rightmost) HF_ST_ bin in A1 and second to lowest (leftmost) HF_ST_ bins in A2 and A3 are significantly enriched. Triangles above the plot indicate the HF_ST_ bins, to which genes known to be under selection belong [29–41]. The figure map was generated using the packages maps v3.1.0 and mapdata v2.2–6 in the R software.

In *dataset D*, we analyzed genetic differentiation between East Asian populations in four areas of different size: the largest one is the wider East Asian area (A1 including A2-A4) and three smaller ones (which we designated as ‘super-clusters’ in this study) are Han Chinese (A2), Hondo (A3) and Ryukyu (A4) areas (Fig 5a). Individuals in three smaller areas (A2, A3 and A4) were genetically distinct, as shown in the HF_ST_ distance between the clusters (Fig 5b) and the EIGENSOFT PCA plot (Fig 5c). In the wider East Asian area A1, where genetic differentiation was measured among three ‘super-clusters’ (i.e., Han Chinese, Hondo and Ryukyu), windows showing positive selection signature in East Asian populations were enriched in high HF_ST_ bins (Fig 5d); in the smaller areas A2 and A3, on the contrary, positively-selected windows were enriched in low HF_ST_ bins. Analogously, the overall distribution of HF_ST_ values among positively-selected windows was higher for A1 and lower for A2, A3 and A4 than that among non-selected windows (*P* < 0.05 by Mann-Whitney test) (Fig 5d). In other words, locus-specific genetic differentiation and positive selection were positively correlated among three ‘super-clusters’ in the wider East Asian area A1, which were geographically distinct to each other, whereas they were inversely correlated among genetic clusters within the individual ‘super-clusters’. The degree of genetic differentiation (HF_ST_) was smaller in the ‘super-clusters’ than in the wider East Asian area A1, while the rank correlation of HF_ST_ was significant (*P* = 1.9×10^−10^) between A1 and Han Chinese (A2) and modest (*P* = 0.029) between Hondo (A3) and Ryukyu (A4) (S6 Fig).

**Fig 6:**
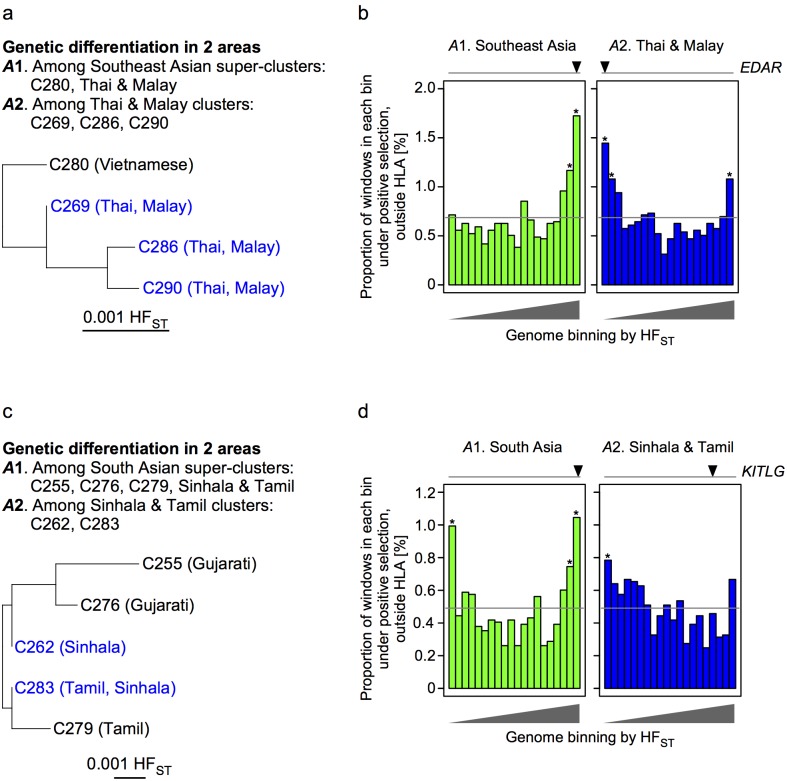
Relation between locus-specific genetic differentiation and positive selection in Southeast Asia and South Asia. **a** and **c**) Neighbor-joining phylogenetic tree of the genetic clusters. **b** and **d**) The proportion of positively selected windows (in vertical axis) is plotted across 20 HF_ST_ bins of 4-SNP windows (in horizontal axis); the average proportion is indicated by a gray line. Asterisk (*) indicates significant enrichment of positively selected windows in a specific bin, compared to the whole-genome average (Fisher’s exact test *P* < 0.01/20). Triangles above the plot indicate the HF_ST_ bins, to which genes known to be under selection belong [29,34–36,39].

We further examined whether the geographic scale-dependent correlation between positive selection and genetic differentiation was detectable in other parts of Asia as well as in East Asia. In Southeast Asian *dataset E*, there were four major genetic clusters, three of which, C269, C286 and C290, were closely related (Fig 6a). In the wider Southeast Asian area A1, positively-selected windows were enriched in high HF_ST_ bins; in the smaller area (A2 including three closely-related clusters), positively-selected windows were also enriched in low HF_ST_ bins. The overall distribution of HF_ST_ values among positively-selected windows was higher for A1 and lower for A2 than that among non-selected windows (*P* < 0.05) (Fig 6b). Similar findings were identified in South Asian *dataset F* (Fig 6c and 6d). Consequently, in all three parts of Asia, positive selection was associated with the reduction of genetic differentiation among closely-related clusters.

To corroborate this, we also measured locus-specific genetic differentiation at 6 genes loci that were known to be under positive selection in Asians (Figs 5d, 6b and 6d, S7 Table). Not all but some of the tested loci, i.e., *EDAR*, *KITLG* and *ALDH2*, supported the geographic scale-dependent effects of positive selection on genetic differentiation. The *EDAR* locus, which is associated with hair and tooth morphology [34–36], was positively selected in East and Southeast Asians of our dataset. In both parts of Asia, HF_ST_ at *EDAR* was in the highest bin for the wider areas (A1) but in the lower bins for the Han Chinese and Thai/Malay areas (A2). Similarly, the *KITLG* locus, which is associated with pigmentation, was positively selected in East and South Asians; HF_ST_ at *KITLG* was in the highest bin for the wider areas (A1) but not so in the smaller areas. The *ALDH2* locus, which is associated with aldehyde metabolism, hypertension and esophageal cancer, was positively selected in East Asians [32,37]; HF_ST_ at *ALDH2* was in the highest bin for the wider area (A1) but not so in the smaller areas. Unexpectedly, the *ABCC11* locus, which is associated with earwax type [38], showed high HF_ST_ among Ryukyu clusters (A4) but not in other areas.

## Discussion

We have investigated a fine-scale genetic structure of the Japanese population by solely using genetic data and have found three significant features of the Japanese population in the present study. First, the Japanese population can be separated into nine clusters in our dataset, showing a marked concordance with geography, which has not been captured in details by previous studies. Second, major components of ancestry profile of Japanese are from the Korean and Han Chinese clusters, the proportion of which differs among Japanese genetic clusters, and for the Japanese as a whole, there are some genetic components from all of the Central, East, Southeast and South Asia. Third, genetic differentiation among the Hondo clusters, which are demographically close but modestly distinct to each other, appears to be caused mostly by genetic drift, whereas genetic differentiation between the Ryukyu and Hondo people, who are socio-geographically distant, has been suggested to be caused partially by positive selection, as previously reported for the *EDAR* and *ABCC11* loci [2,29,33]. In this line, of note is our finding that more generally, in three parts of Asia, the effects of positive selection on genetic differentiation can be in the opposite direction, depending on geographic (and presumably demographic) distantness/closeness between the compared populations; that is, locus-specific genetic differentiation and positive selection are positively correlated between ‘distant’ populations, but positive selection was associated with the reduction of genetic differentiation between ‘close’ populations.

Our analysis expands a previous work on genetic structure of the Japanese population [2] in several aspects. Above all, application of the haplotype-based fineSTRUCTURE method instead of the model-free, SNP genotype-based structure inference (e.g., EIGENSOFT) to genome-wide SNP data has enabled us to divide the Hondo people into multiple fine-scale genetic groups. Our dataset also provides comprehensive coverage of the Hondo people, by including individuals from two of eight districts, the Chugoku (Shimane) and Shikoku (Ehime) districts, which were not included in the previous study [2,3]; individuals from these two regions have turned out to be most diverged among the Hondo people (Fig 1). Moreover, a large number of continental Asian populations are examined to elucidate the ancestry profile and genetic admixture of Japanese populations and have uncovered the presence of some heterogeneity in the ancestry profile of Japanese, even among the Hondo people.

From our widely-sampled continental Asian populations (which consist of 34 populations including the Japanese), we have successfully detected significant evidence for admixture in the Japanese population with clusters C282 (Korean), C284 and C291 (Han Chinese), and C280 (Vietnamese) used as references (*P* < 0.05 for all of the instances in Table 1). Here, an admixture source approximated by a reference of East Asians, e.g., C282, C284 and C291, agrees with the origin of Yayoi people, who are assumed to have come through the Korean Peninsula as the second wave in the dual structure model [1,4–6]. An admixture source approximated by a reference of Southeast Asians is of interest, since the origin of Jomon people has been hypothesized to be in Southeast Asia. Although the detection of C280 as a reference cluster in the ALDER analysis may support such a possibility, it can rather be indirect representation of an East Asian source, considering the findings that C280 (Vietnamese) had high similarity to Han Chinese (Fig 3) and, moreover, other Southeast Asian clusters were not detected as admixture sources. In a phylogenetic analysis of ancient Jomon DNA, Jomon people is shown to form a sister clade to all modern East Asians, indicating a possible early spilt of Jomon people during human migration to East Asia [16]. This agrees with the fact that we detected admixture sources for the Yayoi origin but not for the Jomon origin, despite our extensive sampling of modern Asian populations.

To discern ancestral populations more directly, we performed the ADMIXTURE analysis (Fig 4). Among eight ancestral populations inferred for the Japanese and continental Asians, two East-Asia-wide and one Japan-local ancestral populations were found to contribute to the modern Japanese. In the Japanese, the Hondo people were majorly contributed by the two East-Asia-wide ancestral populations (8% + 49%), whereas the Ryukyu people were majorly contributed by the Japan-local ancestral population (75%). The finding that the Japanese are admixed by wide and local ancestral populations is also reported in a previous ADMIXTURE analysis [42], in which the local ancestral population was represented by the Ainu people that form a deep branch of East Asian diversity. We can speculate that the East-Asia-wide and Japan-local ancestral populations correspond to the Yayoi and Jomon origins.

Our estimate of admixture events in the present study is consistent with a model that has been hypothesized, based on history and archeology but not necessarily on sufficient evidence from genetic data in sample size and autosomal genomic coverage. In the relevant model, it has been speculated that after the Yayoi people migrated to the Japanese archipelagos in the Yayoi period (3,000–1,700 BP), they spread first to the main islands of Japan (Hondo) and later to Okinawa (Ryukyu) [6], leading to admixture with the indigenous people, who are earlier migrants in the Jomon period (>16,000–3,000 BP). Analyzing genetic data, we estimate that the admixture times for the spread are ~50 and ~39 generations ago for *Midland* and *Ryukyu 1* clusters, respectively (Table 1), which agrees with the admixture time and historical events previously reported; that is, [15] estimated 55–58 and 43–44 generations ago for the admixture of the Hondo and Ryukyu people, respectively. The admixture time agrees very well, although the modeling of source populations differed between two studies; the previous study [15] used the Han Chinese and the Ainu people to approximate the two sources. In theory, admixture can take place either with a source newly migrated from the Asian continent at a particular time or gradually between two Japanese populations having different continental Asian ancestry profiles, and the latter scenario is supported by historical events. It is estimated that around the 7th century (1300–1400 years ago), people in the Yamato government (who were admixture of the Yayoi and Jomon people) extended from west to east in the main island of Japan and probably admixed with the aboriginal Emishi people, who were descendants of the Jomon people and lived in the north-east part of the main island. The admixture dating methods based on linkage disequilibrium, such as ALDER, do not detect a series of preceding events (e.g., in the case of people in the Yamato government) but the most recent admixture event [43]. Indeed, the historical records coincide with our estimate that admixture events in the *Midland* cluster were ~50 generations ago (Table 1), which corresponds to year 1250 assuming a 25 year generation time. Another historical event is the migration of people from the main islands of Japan to Okinawa during the proto-Gusku period (11–12th century, 800–1000 years ago) when agriculture was transmitted along with the migrating farmers. This also coincides with our estimate that admixture events in the *Ryukyu 1* cluster were ~39 generations ago (Table 1), corresponding to year 975. To estimate the admixture dating, we and [15] have both used common SNPs on commercial microarrays, yet the dating is unlikely to be affected by the ascertainment bias of SNPs, for the following reason [26]. The ALDER method analyzes how the linkage disequilibrium between a pair of SNPs changes according to the frequency difference of the SNPs in the reference populations. As such, the method is not based on the overall amplitude of linkage disequilibrium, which can be affected by the choice of SNPs.

Another issue of particular note in the present study is the investigation of positive selection and genetic differentiation. Genetic differentiation is caused by genetic drift, natural selection and polygenic adaptation [44]. Positive selection drives global ethnic diversity at many loci, and as such, a measure of locus-specific genetic differentiation such as F_ST_ has previously been used as an indicator of genes under positive selection [45]. We have found that locus-specific genetic differentiation and positive selection are positively correlated between geographically and genetically ‘distant’ populations, but inversely correlated between ‘close’ populations. This was reproducibly detectable in East Asia (Fig 5d), Southeast Asia (Fig 6b) and South Asia (Fig 6d). The 15% of the genome most differentiated in ‘distant’ populations (A1) was enriched for positively-selected regions more than the genome-wide average, whereas there was no enrichment in the corresponding 15% in ‘close’ populations (A2, A3, A4). On the contrary, the 10% of the genome least differentiated in ‘close’ populations was enriched for regions under positive selection, whereas there was no enrichment in the corresponding 10% in ‘distant’ populations.

For a potential mechanism behind the observed geographic scale-dependent relation between locus-specific genetic differentiation and positive selection, we speculate positive selection causing a cline—a gradual change in allele frequencies over an appreciable geographic distance. In this way, positive selection at a locus could accentuate genetic differentiation among ‘distant’ populations, but attenuate genetic differentiation among ‘close’ populations. One scenario is differential selection acting in different parts of a structured population. Such a scenario has been observed in Sardinia island [46], where the frequency of thalassemia allele is high in coasts (where the allele is advantageous in developing resistance to malaria) but low in mountains. Thus, it has been assumed that genetic differentiation was accentuated across the whole island, whereas it was attenuated among towns within the coasts (or within the mountains). Another scenario is an ongoing geographic spread of a positively-selected new variant. One example for this is that the *EDAR* 370Ala allele (rs3827760) specific to Asians was suggested to have a selection coefficient of 0.114 and to have arisen approximately 30,000 years ago in central China, where the current allele frequency is highest [29]. Another example is the *ALDH2* 504Lys allele (rs671) specific to East Asians, whose frequency gradually declines from the peak at Southeast China to the surrounding populations [30–32]. For positively-selected variants that can fit to the second scenario, we may have a better chance to identify that genetic differentiation is accentuated more prominently as populations from the farther locations will be included in the analysis. Compared to the *EDAR* and *ALDH2* SNPs, the *ABCC11* SNP rs17822931 has several different features. It is globally polymorphic; the *ABCC11* 180Arg allele is associated with the absolute latitude consistently but independently in Asian, Native American and European populations, thus showing local adaptation; and the allele was found to be positively selected with a selection coefficient as low as 0.01 in East Asians [33]. These properties could have contributed to the unexpectedly high HF_ST_ among Ryukyu clusters (A4 in Fig 5d). Different from these examples of specific loci, the correlation reported here is a genome-wide property and therefore is deemed to be causative indirectly.

As positive selection assumedly acts on a small proportion of the whole genome, the major driver of genetic differentiation is always random genetic drift. Thus, in the limited genome regions, on which positive selection is acting, people may wonder whether genetic differentiation is accentuated, attenuated, or not affected. Each of the three cases can be visualized in bar graphs in Fig 5d; the bars are higher in high HF_ST_ bins, higher in low HF_ST_ bins, or overall flat, respectively. In this work, although we statistically tested the trends, we did not quantify how much of positive selection contributes to the three cases. One simple way would be to sort the 4-SNPs windows from the genome into three equal-sized bins, and to compare the relative number of windows under positive selection.

Overall, our data suggest that positive selection can accentuate or attenuate locus-specific genetic differentiation between the compared populations in either of the directions, depending on the geographic scale of the investigated area. In principle, large populations among which there is much migration tend to show little genetic differentiation, whereas small populations among which there is little migration tend to be highly differentiated [45]. Also, it is considered that high levels of population (or genetic) differentiation can suggest the action of positive selection with advantageous alleles in one or more populations, i.e., diversifying selection that is often a consequence of local adaptation, whereas lower levels of population differentiation can be considered as the effect of balancing selection that tends to maintain a constant proportion of alleles across all populations, i.e., stabilizing selection [47]. Genetic differentiation can be affected by other demographic factors; for example, admixture may generate a population whose evolutionary process cannot be modeled simply by genetic drift and selection. Although the demographic history of population (e.g., isolation by distance) as well as random genetic drift can influence among-population differences, further investigation from the viewpoint of population genetics is warranted for our finding that the same loci show signatures of both diversifying and stabilizing selection, e.g., HF_ST_ at *EDAR* was in the highest bin for the wider areas (A1) but in the lower bins for the Han Chinese and Thai/Malay areas (A2) (Figs 5d and 6b).

This report is one of the earliest studies on the nation-wide, haplotype-based fine population structure [20,48]. We have found that a haplotype-based clustering method, fineSTRUCTURE, can distinguish people living in different parts of Japan. In the Shimane prefecture, for example, there has turned out to be a dramatic difference in population structure between two cities, which are only 30 km apart (Fig 2). The higher resolution of fineSTRUCTURE compared to EIGENSOFT is due to the use of haplotype sharing instead of SNP sharing, as combining SNP markers into haplotypes improves population structure inference [49]. It has to be noted that both fineSTRUCTURE and EIGENSOFT cannot but sacrifice accuracy to some extent when representing complex or high-dimensional genetic diversity by simplified structures, such as a few discrete clusters or low-dimensional linear space. In the case of fineSTRUCTURE, its resolution of clustering becomes finer as the sample size increases; however, the subdivided clusters may not split hierarchically if the actual genetic diversity is continuous. In fact, although the Shimane population could be subdivided from two large clusters (*Shimane 1* and *2*) to five finer clusters (*Shimane A-E*) when the sample size increased in this study, some of the finer clusters were not strictly a sub-cluster of large cluster (Fig 2b). Despite such limitations, the clustering and dimensionality reduction methods, i.e., fineSTRUCTURE and EIGENSOFT, have been generally used in population genetics as a pragmatic approach [50].

Identification of fine-scale ancestry profile of individuals has substantial implications in medical genetics as well as forensics. In GWAS, population stratification should be measured and carefully controlled. As the sample size of GWAS is expanding to tens or hundreds of thousands of individuals in search of molecular variants with subtle effects on disease, controlling genetic structure becomes more indispensable. As shown in the previous study [2], our data indicate that population stratification in the Hondo people is strong enough to bias GWAS (S3 Table) and not negligible.

Apart from the caveats in the design of GWAS, the improvement of population structure inference is important in the clinical application of human genome sequencing. Resequencing individuals from genetically different populations is thought to help catalogue low-frequency variants, the database of which is useful for sifting rare pathogenic variants from non-pathogenic ones. For rare diseases, knowing that apparently sporadic patients share their ancestry to each other might lead to the discovery of common causal variants, which are private to the tested population. In the analysis for Shimane prefecture, increasing the number of individuals has enabled finer genetic clustering, which corresponds with geography more precisely. This indicates the complexities of genetic structure in real populations. Future work with more populations with larger sample size and wider geographic coverage in Japan and Asia is expected to refine the genetic structure of the Japanese and to advance genetic and evolutionary studies.

## Methods

### Datasets

We used SNP genotype data of eight Japanese populations collected by the Japanese Genome Variation Consortium plus those of 26 other Asian populations. The set of 26 populations consisted of 25 populations studied in the Asian Diversity Project (ADP) [51] and the Yakut population from the Human Genome Diversity Project [24]. The ADP data have been deposited at the European Genome-phenome Archive under the accession number EGAS00001002100. Further, the ADP includes publicly available data of four populations from the HapMap project [52], three populations from the Singapore Genome Variation Project [53] and four populations from the Jorde Lab [54]. The Ainu people were not included in the present study. All individuals were enrolled from the general population. Six datasets were used in accordance with analytical purposes as described later: *dataset A* comprised eight Japanese populations with each involving a randomly selected sample of 200 (a total of 1600 individuals); *dataset B* expanded *dataset A* by 228 in Shimane prefecture (1828 individuals in total); *dataset C* included *dataset A* and continental Asian populations (3928 individuals); *dataset D* included *dataset A* and Han Chinese populations (1864 individuals); *dataset E* included Southeast Asian populations (689 individuals); and *dataset F* included South Asian populations (671 individuals). *Datasets A–C* were used for clustering of Japanese individuals with (*dataset C*) and without (*datasets A* and *B*) reference to other Asian populations. *Datasets D–E* were used for analysis of genetic differentiation, where we chose to include a subset of populations that had been genotyped with Illumina arrays to maximize the number of shared SNPs. See S1 Table for the number of individuals and genotyping arrays in each dataset and S7 Fig for schematic explanation of each dataset. All participants provided written informed consent, and the data was accessed anonymously. Sample enrollment and DNA genotyping were performed in accordance with the ethical standards of the ethical committees of individual participating institutions as stated in the ADP publication [51].

### Quality control of genetic data

We performed quality control (QC) for samples and SNPs in each of the eight Japanese populations in a step-wise manner. First, outlier samples for the number of heterozygous SNPs were removed using the PLINK program (version 1.07) [55]. The PLINK program (option—het) estimates the inbreeding coefficient *F* by comparing the observed versus expected number of heterozygous genotypes. It is likely that excessive heterozygous genotypes, which corresponds to low *F*, are due to genetic admixture or DNA contamination, while deficient heterozygous genotypes and high *F* are due to inbreeding. We removed samples with *F* < –0.02 or *F* > 0.04. Second, an identity-by-state distance was calculated using the PLINK program (options—cluster—neighbour 1 5) for all pairs of samples, where the relevant samples were removed to resolve pairs with extremely high similarity (Z-score > 4) which can be blood relatives; the criterion could detect pairs with identity-by-descent proportion >0.25. Similarly, we filtered pairs with extremely low similarity (Z-score < -4), which can be population outliers; 0–6 samples (1.7 on average) were removed from each Japanese population. Third, we removed outlier samples in genotype similarity using the smartpca program (options numoutevec = 10, numoutlieriter = 5, numoutlierevec = 10, outliersigmathresh = 6) in the EIGENSOFT package (version 5.0.2); 0–4 samples (0.6 on average) were removed from each Japanese population. Fourth, we removed samples with an excess number of rare genotypes using the RHH program (version 0.1) [56]. For the second and third steps, we used a subset of unlinked SNPs, which could be selected by pruning SNPs in linkage disequilibrium using the PLINK program (option—indep 50 5 2). Moreover, in each Japanese population, we removed SNPs showing call rate <1, minor allele frequency (MAF) <0.01 or a Hardy-Weinberg equilibrium *P*-value of <10^−6^. Sample and SNP QC for the other Asian populations had been performed in the ADP. All SNPs were mapped to the positive strand of Human Reference Genome GRCh37.

In the Japanese, the Hanamaki population was assayed with the Affymetrix array, whereas the others were assayed with the Illumina array. Therefore, to increase power in *datasets A*, *B* and *D*, we augmented the Hanamaki population with the genotypes that were imputed using a reference panel of HapMap JPT+CHB+CHD and the BEAGLE software (version 4.r1399) [57]. The imputation quality was *r*^2^ = 1 for 97.6% and *r*^2^≥0.8 for 99.9% of 292,023 imputed SNPs that could attain a posterior probability of genotype call ≥0.67 for all samples and MAF >0.01.

### Clustering of individuals by genetic data

To perform haplotype-based analyses, we first extracted SNPs that had been genotyped for all samples in the corresponding dataset and phased their genotypes using the SHAPEIT program (version 2.r790) [58]. We then computed a coancestry matrix, which could provide a summary information for the extent of haplotype sharing or ancestral relationships among the individuals in the dataset using the ChromoPainter program (which is included in the fineSTRUCTURE program) [19] under estimated -n parameter 120, 115, 102 and -M parameter 0.000159, 0.000164, 0.000621 for *datasets A*, *B* and *C*, respectively. Briefly, each haploid genome of a given individual (who is called a recipient) was reconstructed or ‘painted’ by choosing the most similar haplotype chunks derived from other individuals (called donors) in the dataset. The coancestry matrix *a*_*ij*_ is a square matrix whose (*i*, *j*) element is the number of chunks that the recipient *i* was painted by the donor *j*. Intuitively, the coancestry matrix *a*_*ij*_ counts the number of recombination events that can support individual *i* being most closely related to *j*, thereby giving a natural measure of ancestry sharing. As we noticed the sub-matrix of the coancestry matrix for the Hanamaki population (as both donors and recipients) to be slightly inflated when the imputed genotype was used in *datasets A* and *B*, we adjusted it by a factor of 0.9427 and 0.9310 for *datasets A* and *B*, respectively (S8 Fig). On average, one haplotype chunk included 29, 30 and 10 SNPs for *datasets A*, *B* and *C*, respectively.

Individuals having similar patterns in the coancestry matrix were clustered together using the fineSTRUCTURE program (version 2.0.4) [19] (S9 Fig). The number of clusters was automatically defined while the convergence of Markov chain Monte Carlo (MCMC) process was determined using the Gelman-Rubin diagnostic for two independent runs. The co-clustering of individuals was concordant between the two runs, indicating the consistency of MCMC sampling (S10 Fig).

In case that some individuals had the genomes similar to more than one cluster, they could have been stochastically classified to a cluster different from the one obtained in the MCMC run performed above. We used the number of such ‘intermediate’ individuals to measure the separability of clusters. For each individual, we computed which cluster it would most probably be reassigned [20] under an alternative MCMC run. Let {*A*_*i*_} be the original clustering. In a sampled iteration of the alternative MCMC run, if the individual belongs to a cluster *B*, the probability for being reassigned to cluster *A*_*i*_ is defined as

$$\left|A_{i}\cap B\right|/\sum_{j}\left|A_{j}\cap B\right|.$$ (1)

We then average the above probability over all sampled iterations. The individual is reassigned to the cluster *A*_*i*_ having the largest probability. Two clusters *A*_1_ and *A*_2_ are difficult to separate, if many individuals are reassigned from *A*_1_ to *A*_2_ (or vice versa). We performed nine alternative MCMC runs, and over those runs, we computed the mean and standard deviation of the number of reassigned individuals.

In addition, to compare resolving power of the clustering methods, we performed clustering by the PCA method of subject similarity using the EIGENSOFT program, in which the similarity among individuals was measured by the correlation of the allele doses of SNPs. For example, we used 54,850 unlinked SNPs in *dataset A*, which were selected by pruning SNPs in linkage disequilibrium in the way as stated above.

To compare the current study with the Japanese population in the BioBank Japan study [2], the individuals in the current study (*dataset A*) were classified according to the principal components for the BioBank Japan population, using the software by [22].

We performed simulations to estimate the potential impact of genetic differentiation in the Japanese on GWAS. Two groups of genetic clusters were compared as cases and controls in the setting of a GWAS for the SNPs in *dataset A* using the PLINK program (option—assoc). The inflation of significance at the median of trend-test *P*-value for the SNPs is represented as *λ*_GC_ according to [23].

### Measurement of genetic differentiation

We measured genetic differentiation by haplotypic F_ST_ (HF_ST_) among clusters that were defined by the haplotype-based fineSTRUCTURE analysis. The genome was partitioned into windows of four SNPs, in each of which locus-specific HF_ST_ for haplotypes was calculated using the pegas library (version 0.8) [59] of the R software (version 3.1.2) according to the formulae by [60]. For each pair of clusters, population-specific HF_ST_ was calculated by averaging the numerator and denominator of locus-specific HF_ST_ over all windows in the genome and taking the ratio of their averages [61]. The neighbor-joining phylogenetic trees of clusters, based on distances defined by population-specific HF_ST_, were computed using the ape package (version 3.2) [62] of the R software and plotted using the FigTree program (version 1.4.2).

### Ancestry profile of Japanese clusters

We computed the ancestry profile of each Japanese cluster analogously to the study by [20]. We used the ChromoPainter (version 2) [19] to compute the coancestry matrix (based on haplotype chunk-lengths) for all the Japanese and continental Asians (*dataset C*) using non-Japanese populations as donors. Estimated -n and -M parameters for this analysis was 124 and 0.000351, respectively.

For each cluster (either Japanese or continental Asian), we extracted rows of the coancestry matrix, where recipients were individuals in the relevant cluster, and computed the average of rows, which can be regarded as a characteristic row for the cluster. For a Japanese cluster *P*, we computed a vector *Y*_*P*_ by first extracting rows of the coancestry matrix for recipients in the cluster *P* and then averaging over the corresponding rows. Analogously, for a continental Asian cluster *g* (with ≥8 individuals), we computed a vector *X*_*g*_ by first extracting rows for recipients in the cluster *g* and then averaging over the corresponding rows.

The vector *Y*_*P*_ itself does not represent an ancestry profile, because it is affected by sample size difference among clusters and incomplete lineage sorting. Instead, the ancestry profile of Japanese cluster *P* is obtainable by approximating the vector *Y*_*P*_ with a mixture of the vectors *X*_*g*_’s for the continental Asian clusters, where the weights in the mixture represent the contributions of the continental Asian cluster. Specifically, we inferred the ancestry profile (*β*_1_, *β*_2_,⋯,*β*_*G*_) by solving a non-negative linear least squares with the nnls package (version 1.4) [63] of the R software,

$$Y_{P}=\beta_{1}X_{1}+\beta_{2}X_{2}+\cdots +\beta_{G}X_{G},$$ (2)

under *β*_*g*_ ≥ 0 and *β*_1_ + *β*_2_ + ⋯ *β*_*G*_ = 1, where the coefficient *β*_*g*_ represents the contribution of the continental Asian cluster *g* to the Japanese cluster *P*.

We also performed 1000 bootstrap resamplings to estimate the 95% confidence interval of *β*_*g*_’s. For each autosome (e.g., chromosome 22), we resampled a homologous pair of chromosomes of a given individual from the pool of pairs for the individuals in the same Japanese cluster by replacement. An individual was allowed to have >1 chromosomes (e.g., chromosomes 21 and 22) originally belonging to different individuals. In practice, the coancestry matrix for each chromosome of the original dataset was computed, the rows for the individuals in that cluster were resampled, and the matrices thus resampled were added over the autosomes to yield a final coancestry matrix.

To test if the contribution of a continental Asian cluster differs between Japanese clusters, we performed bootstrap hypothesis testing [64] by using the bootstrap resampling described in the previous paragraph. For a pair of Japanese clusters, let the observed contributions be *β*, *γ* and the *i*-th bootstrap resample be *β*^(*i*)^, *γ*^(*i*)^. From the probability distribution for the difference of contributions, under the null hypothesis of equal contribution, we obtained samples

$$x^{\left(i\right)}=\left(\beta^{\left(i\right)}-\gamma^{\left(i\right)}\right)-\left(\beta -\gamma \right),\;\left(i=1,\cdots ,1000\right).$$ (3)

We counted the proportion of *x*^(*i*)^ with a value more extreme than the observed difference, *β* − *γ*, and doubled the proportion to obtain the two-sided *P*-value. The added contribution from multiple continental Asian clusters can be tested in the same way by assigning the sum of contributions to *β*, *γ*, *β*^(*i*)^, *γ*^(*i*)^.

### Dissection of genetic admixture

To test whether the Japanese population was formed by admixture of multiple Asian populations, we measured admixture-induced linkage disequilibrium, which occurs between two SNPs having different allele frequencies in the source populations, using the ALDER program (version 1.03). In this program, the allele frequency information in the two source populations is approximated by a pair of reference populations, and the admixed population itself can serve as one reference. We used continental Asian clusters with ≥8 individuals as references. For each Japanese cluster, we investigated genetic admixture by using itself as one reference and each of the continental Asian clusters as the other reference. To correct for multiple testing, the ALDER program estimated the effective number of continental Asian populations as 12 and statistical significance should be adjusted accordingly; that is, *P*-values were multiplied by 12 for testing a pair of each Japanese cluster and each of the continental Asian clusters as references. The ALDER program could estimate the timing but not the proportion of admixture.

To test if the timing of admixture agrees among a group of Japanese clusters, we performed meta-analysis of the estimated times and computed the *P*-value for heterogeneity using the rmeta library (version 2.16) of the R software.

To directly model ancestral populations of the Japanese and continental Asians (*dataset C*), we performed unsupervised genetic clustering using the ADMIXTURE program (version 1.3.0). With the parameter *K* for the number of ancestral populations ranging from 1 to 10, we performed five runs each using different random seeds. We chose *K* = 8 as the optimal number of ancestral populations, which attained the lowest cross-validation error. From the runs for *K* = 8, which resulted in log-likelihood differing only by <0.01, we chose the run with the largest log-likelihood.

### Examination of positive selection

We further tested whether chromosomal segments showing prominent locus-specific genetic differentiation among the populations could correspond with those undergoing positive selection. For this, we used three datasets: *dataset D* combining Japanese and Han Chinese, *dataset E* for Southeast Asian and *dataset F* for South Asian populations (S1 Table). For the three dataset, the genome was partitioned into 26,344, 114,859 and 152,993 windows of four SNPs, respectively; we removed outlier windows with regards to length (or width), which diverged more than three times the standard deviation in the log(bp) or log(cM) scale. Locus-specific genetic differentiation was measured by HF_ST_ among the relevant clusters. To estimate HF_ST_ accurately, we restricted the analysis to clusters including ≥42 individuals. We chose chromosomal regions that had been detected to be under positive selection in at least one population in the respective dataset (S8, S9 and S10 Tables) by attaining empirical score < 0.01 in the HaploPS program (version 1.0) in ADP study; the HaploPS scan could detect haplotypes with excess frequency in proportion to their length, although it was performed within a single population and did not use information for inter-population differences. The power of HaploPS is higher than that of iHS and XP-EHH [28], e.g., for a selection coefficient of 0.01, power is 80–90% for an allele frequency range of 0.2–0.9. In the present study, we excluded the extended HLA region from the analysis, because it is known to be highly differentiated and under substantial selection. We also examined 5 target loci (*EDAR*, *KITLG*, *ALDH2*, *ADH1B* and *ABCC11*), which are known or reported to be under positive selection in Asians. Since HF_ST_ takes real values at the four-SNP windows and inclusion in positively selected regions is a 0/1 property, the correlation between locus-specific HF_ST_ and positive selection was tested by Mann-Whitney test.

## Supporting information

S1 Fig EIGENSOFT analysis of Japanese populations. Individuals are plotted by the first two principal components. Nine clusters with 10 or more individuals, which are defined by the fineSTRUCTURE method, are highlighted by different colors. In the EIGENSOFT analysis, the Okinawa people as a whole are separated from the others (Hondo people), while the four Ryukyu clusters (*Ryukyu* 1–4) in the Okinawa people tend to overlap and are indistinguishable to each other. The clusters of Hondo people separate neither by the third to tenth principal components.
(PDF)

S2 Fig Comparison of the current study with the Japanese populations in the BioBank Japan study. Individuals in the current study were classified by the principal components computed for the BioBank Japan populations by using the software by Kumasaka et al. [J. Hum. Genet. 55:525]. **a**) The two studies completely agreed on the classification between Hondo clusters and Ryukyu clusters. **b**) Distribution of BioBank Japan individuals according to the first two principal components are plotted as gray clouds of points. **c**) Individuals in the current study are overlaid as colored points.
(PDF)

S3 Fig Genetic differentiation measured by HF_ST_ between the Japanese and continental Asian genetic clusters (a) with close-up views for the Hondo (b) and Ryukyu (c) clusters, and the neighbor-joining phylogenetic tree based on the HF_ST_ distance (d). The dotted lines are added to indicate the cluster names of tree nodes, thus do not represent branches of the phylogenetic tree. See S5 Table for the values of HF_ST_.
(PDF)

S4 Fig Comparison of admixture time among Japanese clusters. The admixture time was estimated using the 1-reference ALDER test (Table 1). The squares and whiskers represent the estimate and SE of the admixture time for single clusters. A diamond represents the meta-analysis of a group of clusters; the center and width of the diamond indicate the combined estimate and SE. The decimal beside the diamond is the *P*-value for heterogeneity of admixture times among the clusters. There was no heterogeneity among the clusters in Hondo or among the clusters in Ryukyu (*P*_het_ > 0.05). However, when all Japanese clusters were combined, there was significant heterogeneity in admixture time (*P*_het_ < 0.05 when the reference population is C284 or C291).
(PDF)

S5 Fig Coancestry matrices for the case where all chromosomes data are combined (a) and for the cases where individual chromosome data are analyzed separately for chromosomes 1 to 6 (b). The names of 9 clusters are depicted to the left of matrix (a), and the numbers of SNPs used for analysis are shown in parentheses at the top of each matrix (b). The former matrix is the sum of the matrices for all autosomes. The values for individuals in the same genetic cluster are averaged. The patterns of the matrices are almost consistent across the chromosomes demonstrated in the figure.
(PDF)

S6 Fig Comparison of HF_ST_ ranking among the four East Asian areas. Correlations of HF_ST_ ranking among the four East Asian areas are shown in the left table. For the 4-SNPs windows at top 5% of HF_ST_ in area A1, the distribution of HF_ST_ values in the other three areas are shown in the right plot.
(PDF)

S7 Fig Schematic representation of datasets used in the study. The figure maps were generated using the packages maps v2.3–9 and mapdata v2.2–3 in the R software.
(PDF)

S8 Fig Effect on coancestry matrix of the usage of imputed genotypes for the Hanamaki population. For *datasets A* and *B*, we compared the coancestry matrices computed using the imputed genotypes or not. For seven Japanese populations, the average number of haplotype chunks donated from a donor to a recipient in the same population were plotted. The Okinawa population was not included in this analysis, because it was genetically distinct from the other Japanese populations. The average number of chunks for the Hanamaki population (red circle) is slightly inflated when the imputed genotypes are used. The black line was fitted for the remaining six populations. The circle for the Hanamaki population would lie on the fitted line, if the y-coordinate is multiplied by 0.9427 or 0.9310 for *datasets A* and *B*, respectively. To the coancestry matrices based on the imputed genotypes, we applied the corresponding scaling to the sub-matrix of the Hanamaki population, and used them in the downstream analyses. The scaling canceled the inflation caused by imputation inaccuracy.
(PDF)

S9 Fig Dendrogram of fineSTRUCTURE clustering for *dataset A*. There are 1600 tip nodes corresponding to individuals, 1599 internal nodes, and 3198 edges in the tree. The tip nodes are aligned on the right end from top to bottom, and colored according to genetic clusters. Most of the edges near the tip nodes are very short and invisibly condensed in the right end of the figure. The tree was drawn using the FinestructureRcode that accompanies the fineSTRUCTURE program.
(PDF)

S10 Fig A pairwise coincidence matrix for two independent runs of fineSTRUCTURE clustering for dataset A. The (*i*, *j*) dot represents the posterior probability of the *i*-th and *j*-th individuals belonging to the same cluster. The dot is colored by the probability according to the scale shown in the right to the plot. The lower-left and upper-right parts of the plot represent two independent runs of Markov chain. Almost symmetrical patterns with the diagonal line as a symmetrical axis indicate the stability of fineSTRUCTURE clustering.
(PDF)

S1 Table Study populations and genotyping arrays. (XLSX)

S2 Table Reassignment of individuals to genetic clusters in an alternative MCMC run. (XLSX)

S3 Table Genetic differentiation between Japanese genetic clusters measured by *λ*_GC_. (XLSX)

S4 Table Continental clusters and their proportions in the ancestry profiles of Japanese clusters. (XLSX)

S5 Table Genetic differentiation between genetic clusters measured by population-specific HF_ST_. (XLSX)

S6 Table Correlation coefficients between coancestry matrices computed for single chromosomes in *dataset A*. (XLSX)

S7 Table Locus-specific genetic differentiation at functional loci under positive selection in Asians. (XLSX)

S8 Table Chromosomal regions detected to be positively selected in East Asian populations in *dataset D*. (XLSX)

S9 Table Chromosomal regions detected to be positively selected in Southeast Asian populations in *dataset E*. (XLSX)

S10 Table Chromosomal regions detected to be positively selected in South Asian populations in *dataset F*. (XLSX)
