# Supplementary material for: The fine-scale genetic structure and evolution of the Japanese population
Source: PLoS One. 2017 Nov 1;12(11):e0185487. doi: 10.1371/journal.pone.0185487 (PMC5665431; doi:10.1371/journal.pone.0185487)
Supplement: S1 Fig — Individuals are plotted by the first two principal components. Nine clusters with 10 or more individuals, which are defined by the fineSTRUCTURE method, are highlighted by different colors. In the EIGENSOFT analysis, the Okinawa people as a whole are separated from the others (Hondo people), while the four Ryukyu clusters (Ryukyu 1–4) in the Okinawa people tend to overlap and are indistinguishable to each other. The clusters of Hondo people separate neither by the third to tenth principal components. (PDF) [file pone.0185487.s001.pdf]

Figure S1
EIGENSOFT analysis of Japanese populations. Individuals are plotted by the first two principal components. Nine clusters with 10 or more individuals, which are defined by the fineSTRUCTURE method, are highlighted by different colors. In the EIGENSOFT analysis, the Okinawa people as a whole are separated from the others (Hondo people), while the four Ryukyu clusters (*Ryukyu 1-4*) in the Okinawa people tend to overlap and are indistinguishable to each other. The clusters of Hondo people separate neither by the third to tenth principal components.

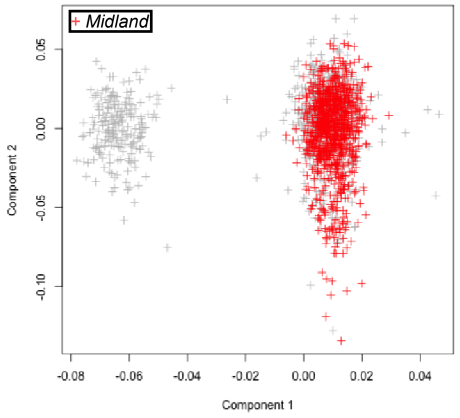

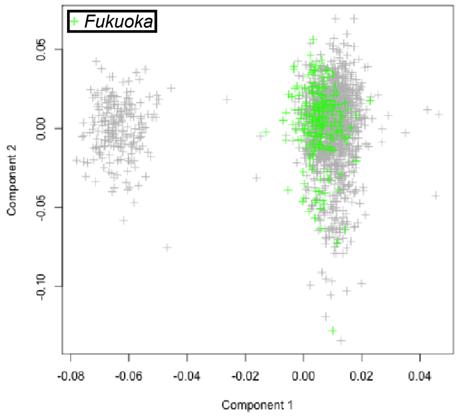

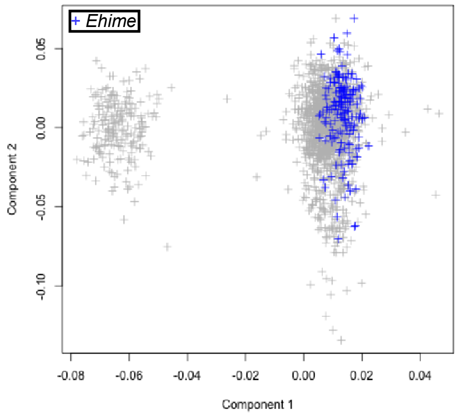

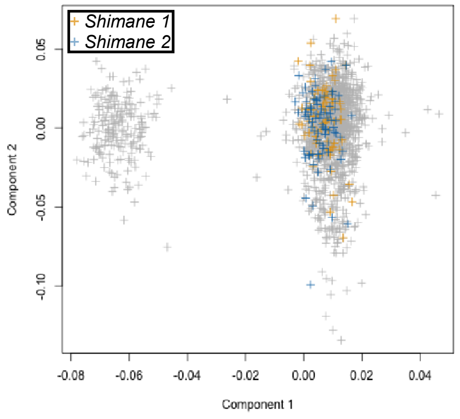

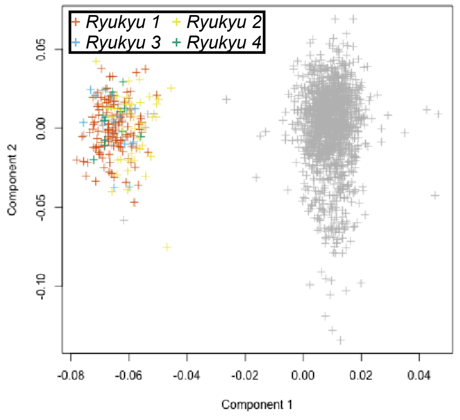

A. Distribution of principal component values in each genetic cluster.

Genetic cluster	Component 1		Component 2	
	Mean	SD	Mean	SD
Midland (n=840)	0.0099	0.0048	-0.0026	0.0267
Fukuoka (n=197)	0.0054	0.0051	0.0019	0.0248
Ehime (n=158)	0.0133	0.0035	0.0083	0.0239
Shimane 1 (n=77)	0.0072	0.0042	0.0053	0.0231
Shimane 2 (n=82)	0.0058	0.0042	0.0009	0.0229
Ryukyu 1 (n=116)	-0.0652	0.0054	-0.0007	0.0167
Ryukyu 2 (n=42)	-0.0583	0.0055	-0.0011	0.0230
Ryukyu 3 (n=13)	-0.0652	0.0057	0.0010	0.0200
Ryukyu 4 (n=13)	-0.0656	0.0044	0.0057	0.0147

B. Statistical significance for difference in average principal component values between Hondo clusters (ANOVA P-value).

Pair of genetic clusters	Component 1	Component 2
Midland vs *Fukuoka*	8.7E-30	0.031
Midland vs *Ehime*	5.6E-17	1.7E-06
Midland vs *Shimane 1*	1.6E-06	0.012
Midland vs *Shimane 2*	1.7E-13	0.25
Fukuoka vs *Ehime*	2.4E-46	0.014
Fukuoka vs *Shimane 1*	7.3E-03	0.29
Fukuoka vs *Shimane 2*	0.55	0.76
Ehime vs *Shimane 1*	2.7E-25	0.36
Ehime vs *Shimane 2*	2.8E-35	0.0214
Shimane 1 vs *Shimane 2*	0.040	0.23

C. Statistical significance for difference in average principal component values between Ryukyu clusters (ANOVA P-value).

Pair of genetic clusters	Component 1	Component 2
Ryukyu 1 vs *Ryukyu 2*	6.6E-11	0.89
Ryukyu 1 vs *Ryukyu 3*	0.97	0.73
Ryukyu 1 vs *Ryukyu 4*	0.80	0.19
Ryukyu 2 vs *Ryukyu 3*	3.1E-04	0.76
Ryukyu 2 vs *Ryukyu 4*	6.5E-05	0.32
Ryukyu 3 vs *Ryukyu 4*	0.82	0.51
