# Supplementary material for: The fine-scale genetic structure and evolution of the Japanese population
Source: PLoS One. 2017 Nov 1;12(11):e0185487. doi: 10.1371/journal.pone.0185487 (PMC5665431; doi:10.1371/journal.pone.0185487)

Figure S2

Comparison of the current study with the Japanese populations in the BioBank Japan study. Individuals in the current study were classified by the principal components computed for the BioBank Japan populations by using the software by Kumasaka et al. [J. Hum. Genet. 55:525]. **a**) The two studies completely agreed on the classification between Hondo clusters and Ryukyu clusters. **b**) Distribution of BioBank Japan individuals according to the first two principal components are plotted as gray clouds of points. **c**) Individuals in the current study are overlaid as colored points.

a

			Assigned geographical regions according to PCA of BioBank Japan study				
			Hondo				Ryukyu
			Tohoku	Kanto	Kinki	Kyushu	Okinawa
Japanese clusters in this study	Hondo	*Midland* (n=840)	2	730	108	0	0
		Fukuoka (n=198)	0	156	39	3	0
		Ehime (n=158)	0	123	35	0	0
		Shimane 1 (n=77)	0	77	0	0	0
		Shimane 2 (n=82)	0	81	1	0	0
	Ryukyu	*Ryukyu 1* (n=116)	0	0	0	0	116
		Ryukyu 2 (n=42)	0	0	0	0	42
		Ryukyu 3 (n=13)	0	0	0	0	13
		Ryukyu 4 (n=13)	0	0	0	0	13

b

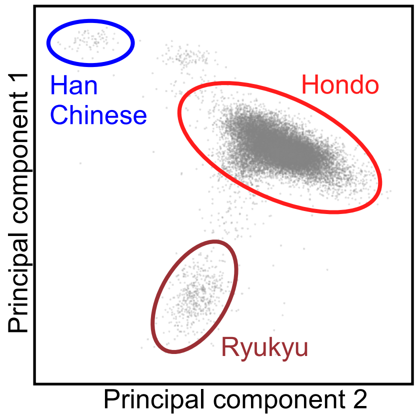

c

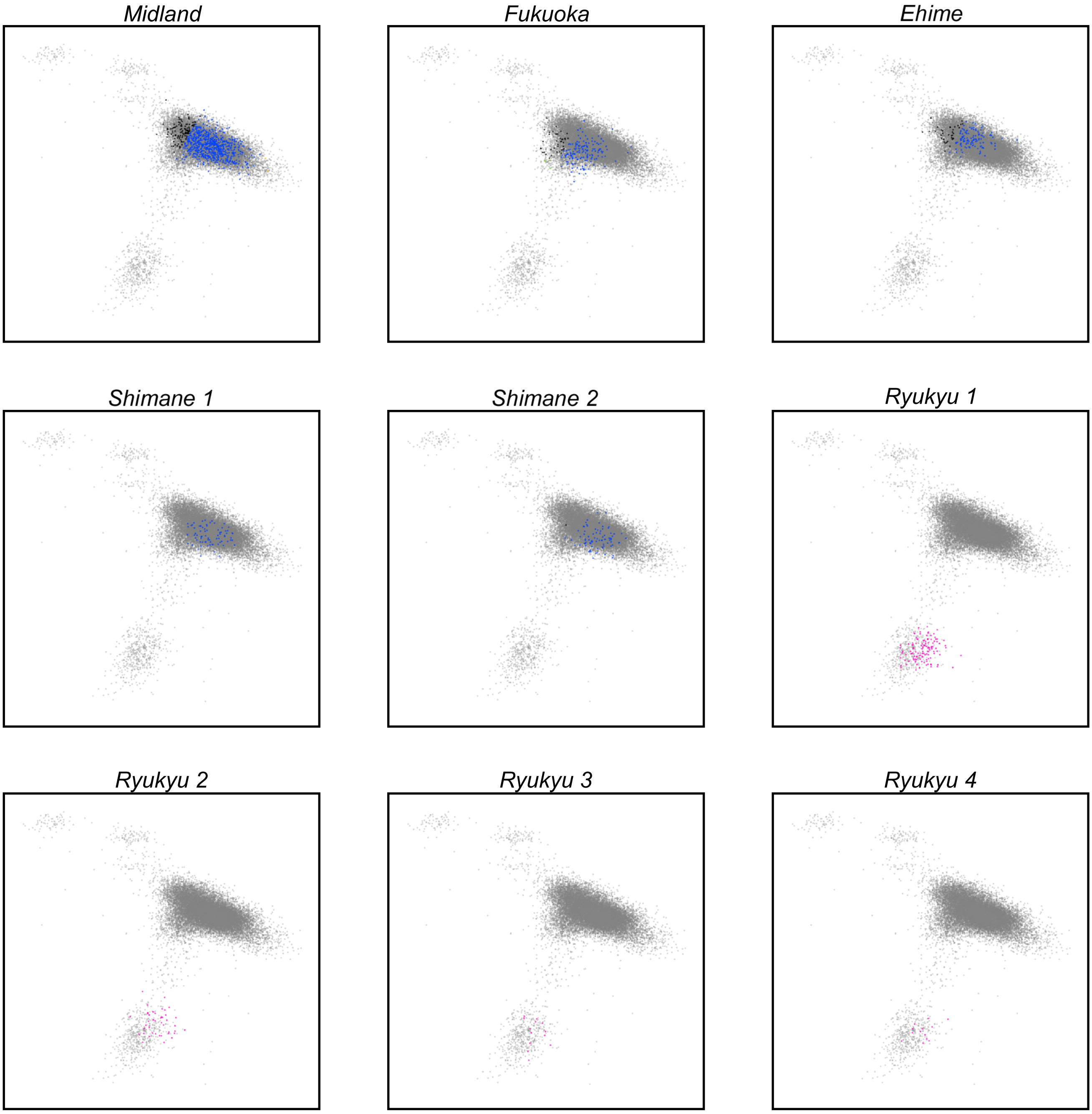

Supplement: S2 Fig — Individuals in the current study were classified by the principal components computed for the BioBank Japan populations by using the software by Kumasaka et al. [J. Hum. Genet. 55:525]. a) The two studies completely agreed on the classification between Hondo clusters and Ryukyu clusters. b) Distribution of BioBank Japan individuals according to the first two principal components are plotted as gray clouds of points. c) Individuals in the current study are overlaid as colored points. (PDF) [file pone.0185487.s002.pdf]
