# Supplementary figures and images for: The fine-scale genetic structure and evolution of the Japanese population
Source: PLoS One. 2017 Nov 1;12(11):e0185487. doi: 10.1371/journal.pone.0185487 (PMC5665431; doi:10.1371/journal.pone.0185487)

Figure S3

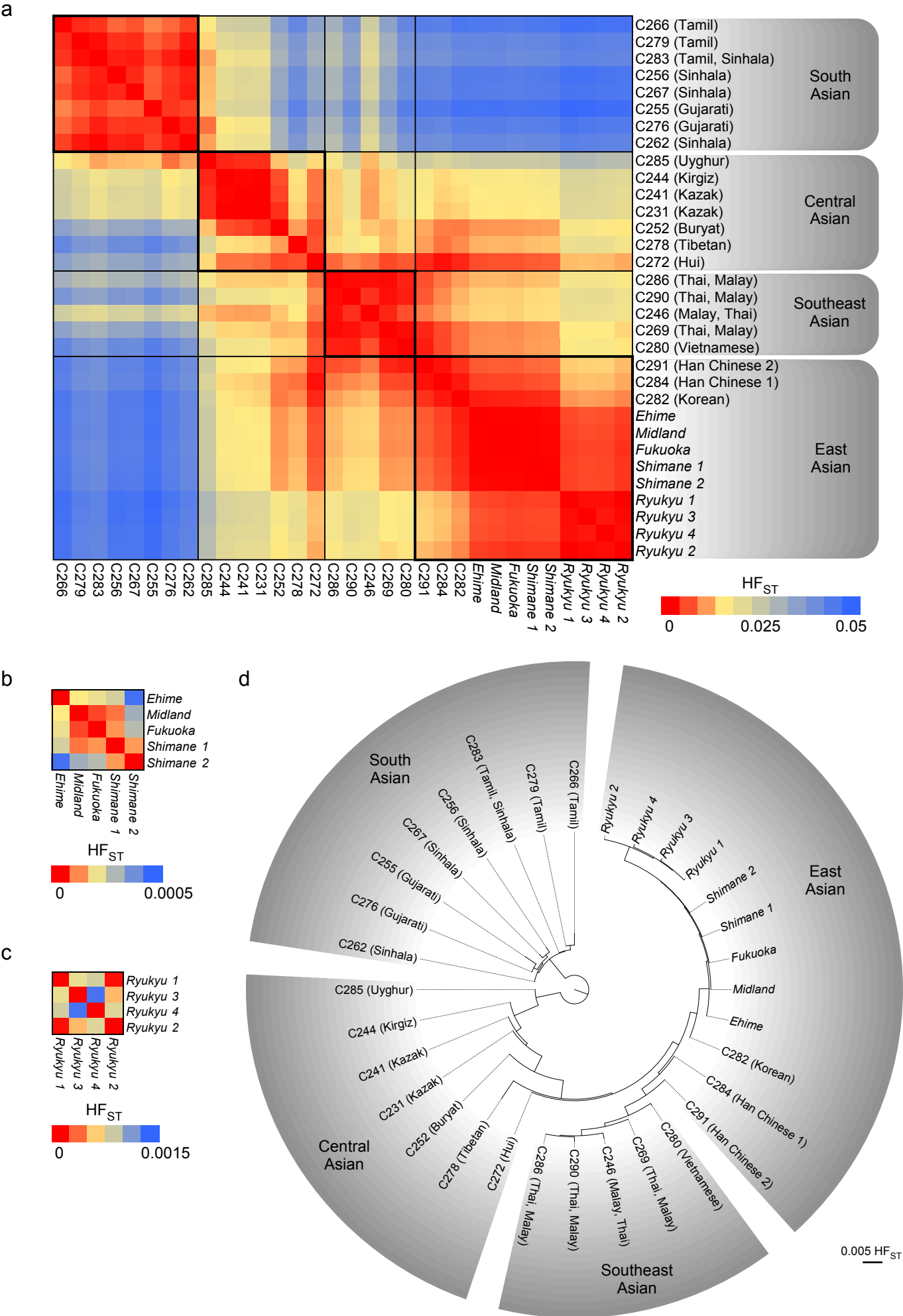

Supplement: S3 Fig — The dotted lines are added to indicate the cluster names of tree nodes, thus do not represent branches of the phylogenetic tree. See S5 Table for the values of HFST. (PDF) [file pone.0185487.s003.pdf]
