# Supplementary material for: The fine-scale genetic structure and evolution of the Japanese population
Source: PLoS One. 2017 Nov 1;12(11):e0185487. doi: 10.1371/journal.pone.0185487 (PMC5665431; doi:10.1371/journal.pone.0185487)

Figure S4

Comparison of admixture time among Japanese clusters. The admixture time was estimated using the 1-reference ALDER test (Table 1). The squares and whiskers represent the estimate and SE of the admixture time for single clusters. A diamond represents the meta-analysis of a group of clusters; the center and width of the diamond indicate the combined estimate and SE. The decimal beside the diamond is the *P*-value for heterogeneity of admixture times among the clusters. There was no heterogeneity among the clusters in Hondo or among the clusters in Ryukyu ($P_{het} > 0.05$). However, when all Japanese clusters were combined, there was significant heterogeneity in admixture time ($P_{het} < 0.05$ when the reference population is C284 or C291).

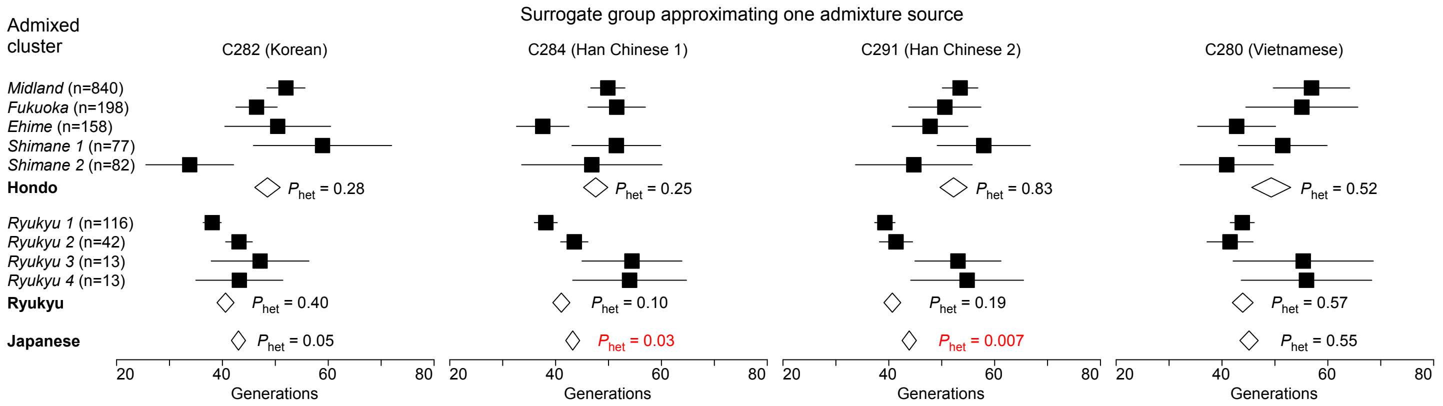

Supplement: S4 Fig — The admixture time was estimated using the 1-reference ALDER test (Table 1). The squares and whiskers represent the estimate and SE of the admixture time for single clusters. A diamond represents the meta-analysis of a group of clusters; the center and width of the diamond indicate the combined estimate and SE. The decimal beside the diamond is the P-value for heterogeneity of admixture times among the clusters. There was no heterogeneity among the clusters in Hondo or among the clusters in Ryukyu (Phet > 0.05). However, when all Japanese clusters were combined, there was significant heterogeneity in admixture time (Phet < 0.05 when the reference population is C284 or C291). (PDF) [file pone.0185487.s004.pdf]
