# Supplementary material for: The fine-scale genetic structure and evolution of the Japanese population
Source: PLoS One. 2017 Nov 1;12(11):e0185487. doi: 10.1371/journal.pone.0185487 (PMC5665431; doi:10.1371/journal.pone.0185487)

Figure S5

Coancestry matrices for the case where all chromosomes data are combined (**a**) and for the cases where individual chromosome data are analyzed separately for chromosomes 1 to 6 (**b**); the names of 9 clusters are depicted to the left of matrix (**a**), and the numbers of SNPs used for analysis are shown in parentheses at the top of each matrix (**b**). The former matrix is the sum of the matrices for all autosomes. The values for individuals in the same genetic cluster are averaged. The patterns of the matrices are almost consistent across the chromosomes demonstrated in the figure.

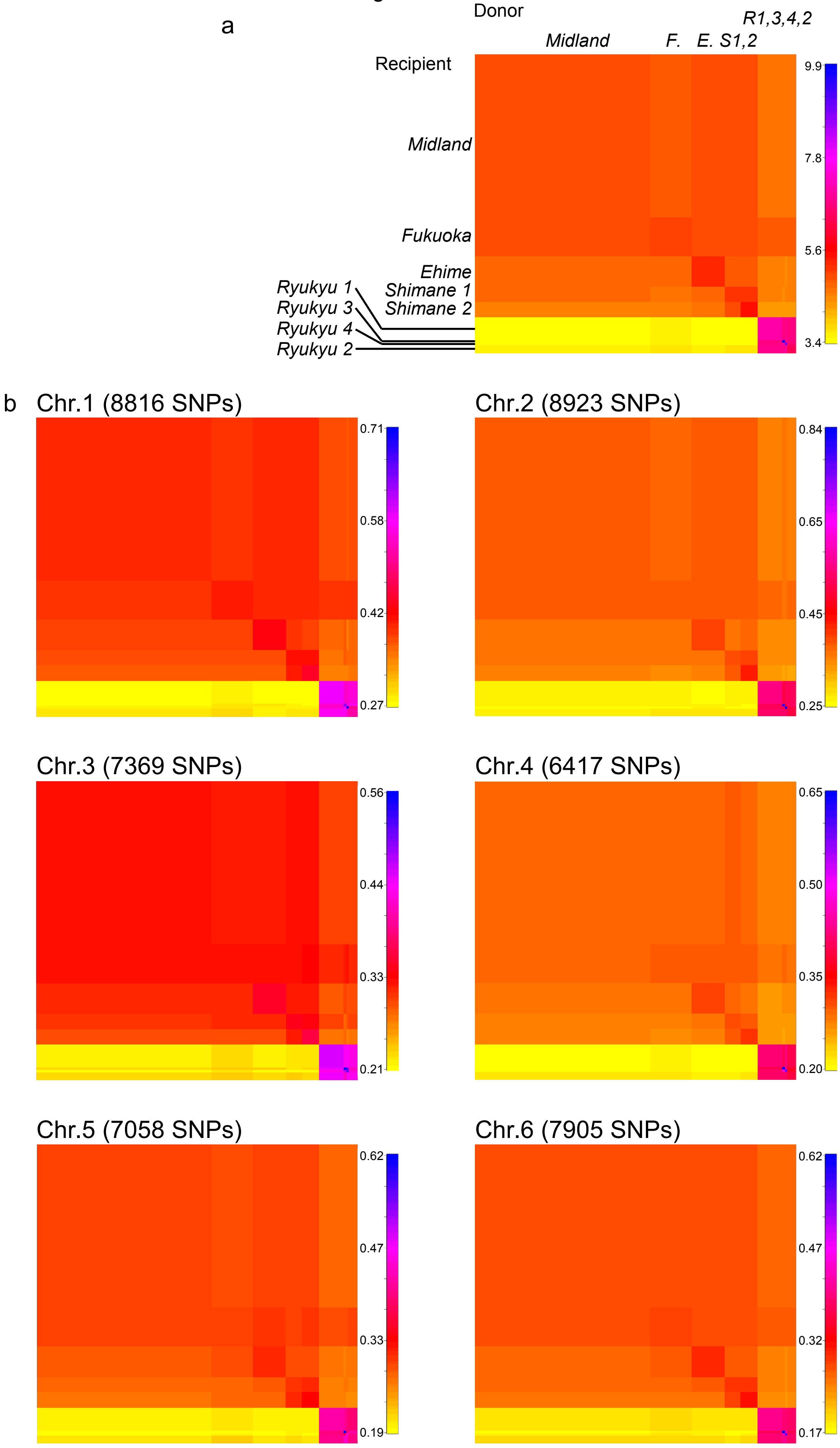

Supplement: S5 Fig — The names of 9 clusters are depicted to the left of matrix (a), and the numbers of SNPs used for analysis are shown in parentheses at the top of each matrix (b). The former matrix is the sum of the matrices for all autosomes. The values for individuals in the same genetic cluster are averaged. The patterns of the matrices are almost consistent across the chromosomes demonstrated in the figure. (PDF) [file pone.0185487.s005.pdf]
