# Supplementary material for: The fine-scale genetic structure and evolution of the Japanese population
Source: PLoS One. 2017 Nov 1;12(11):e0185487. doi: 10.1371/journal.pone.0185487 (PMC5665431; doi:10.1371/journal.pone.0185487)

Figure S6

Correlations of HF_{ST} ranking among the four East Asian areas are shown in the left table. For the 4-SNPs windows at top 5% of HF_{ST} in area A1, the distribution of HF_{ST} values in the other three areas are shown in the right plot.

Rank correlation of HF_{ST}

	*A*2	*A*3	*A*4
*A*1	0.0391 (*P*=1.9e-10)	0.0075 (*P*=0.22)	-0.0064 (*P*=0.29)
*A*2		0.0062 (*P*=0.31)	0.0022 (*P*=0.71)
*A*3			0.0013 (*P*=0.029)

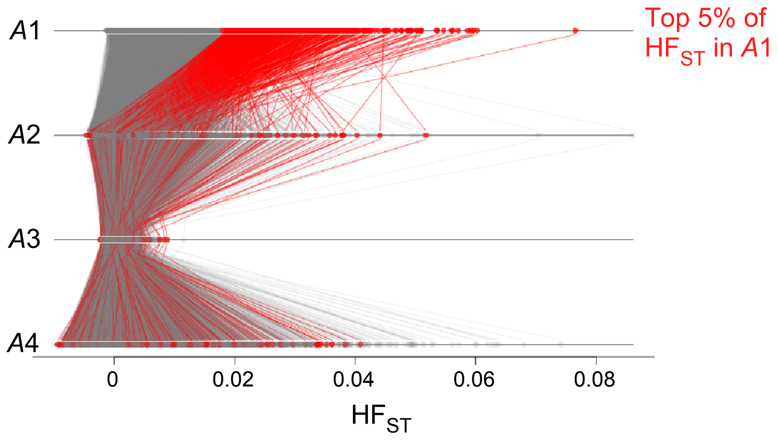

Supplement: S6 Fig — Correlations of HFST ranking among the four East Asian areas are shown in the left table. For the 4-SNPs windows at top 5% of HFST in area A1, the distribution of HFST values in the other three areas are shown in the right plot. (PDF) [file pone.0185487.s006.pdf]
