# Supplementary material for: The fine-scale genetic structure and evolution of the Japanese population
Source: PLoS One. 2017 Nov 1;12(11):e0185487. doi: 10.1371/journal.pone.0185487 (PMC5665431; doi:10.1371/journal.pone.0185487)

Figure S7
Schematic representation of datasets used in the study. The figure maps were generated using the packages maps v2.3-9 and mapdata v2.2-3 in the R software.

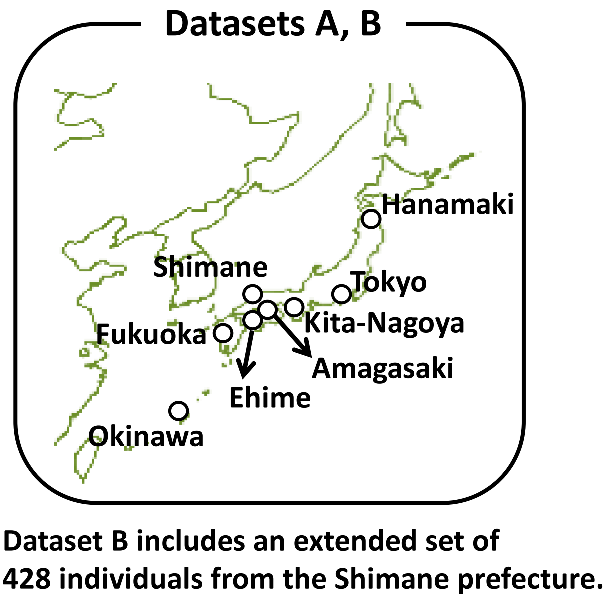

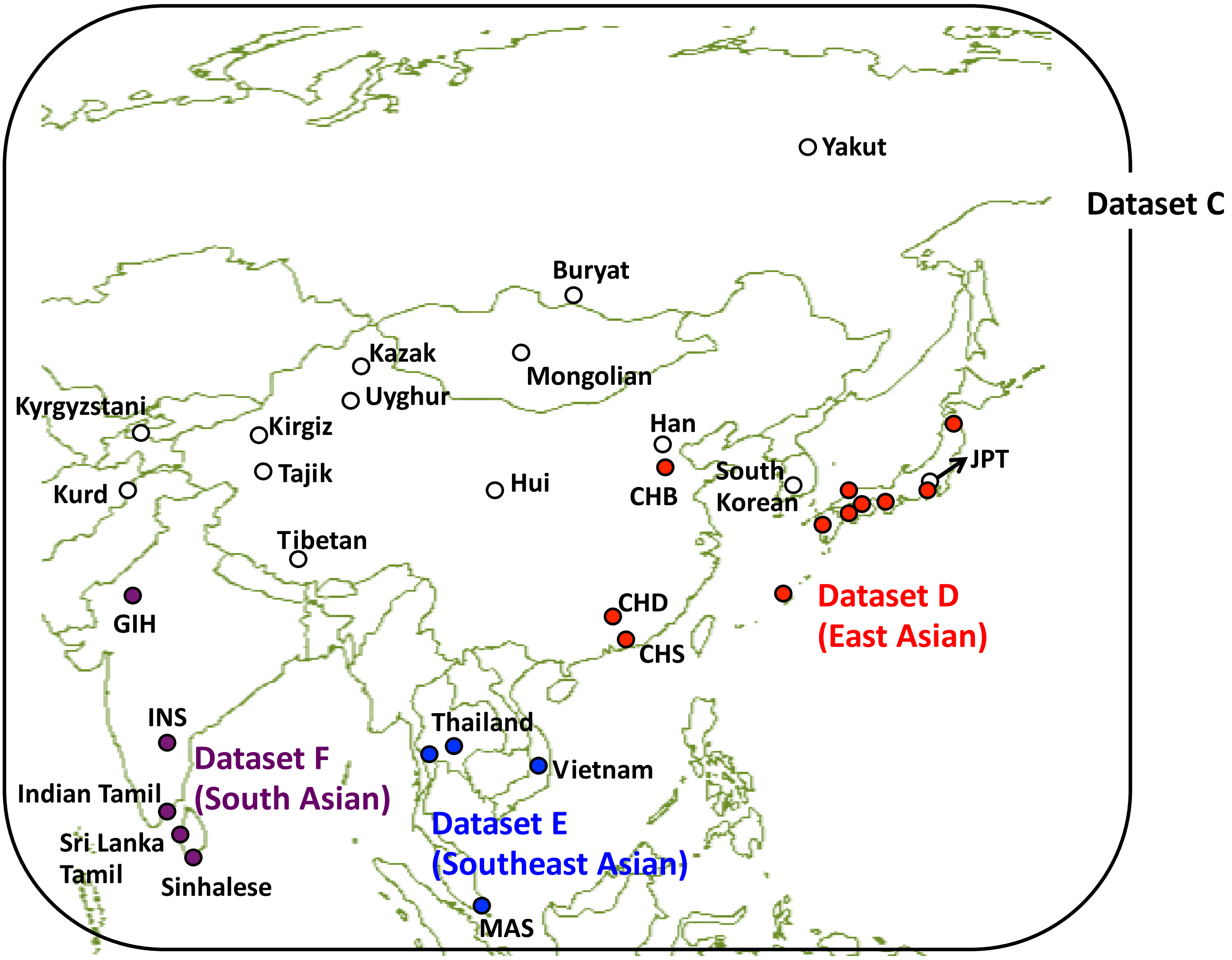

Supplement: S7 Fig — The figure maps were generated using the packages maps v2.3–9 and mapdata v2.2–3 in the R software. (PDF) [file pone.0185487.s007.pdf]
