# Supplementary material for: The fine-scale genetic structure and evolution of the Japanese population
Source: PLoS One. 2017 Nov 1;12(11):e0185487. doi: 10.1371/journal.pone.0185487 (PMC5665431; doi:10.1371/journal.pone.0185487)

Figure S8
Effect on coancestry matrix of the usage of imputed genotypes for the Hanamaki population. For *datasets A* and *B*, we compared the coancestry matrices computed using the imputed genotypes or not. For seven Japanese populations, the average number of haplotype chunks donated from a donor to a recipient in the same population were plotted. The Okinawa population was not included in this analysis, because it was genetically distinct from the other Japanese populations. The average number of chunks for the Hanamaki population (red circle) is slightly inflated when the imputed genotypes are used. The black line was fitted for the remaining six populations. The circle for the Hanamaki population would lie on the fitted line, if the y-coordinate is multiplied by 0.9427 or 0.9310 for *datasets A* and *B*, respectively. To the coancestry matrix based on the imputed genotypes, we applied the corresponding scaling to the sub-matrix of the Hanamaki population, and used it in the downstream analyses. The scaling canceled the inflation caused by imputation inaccuracy.

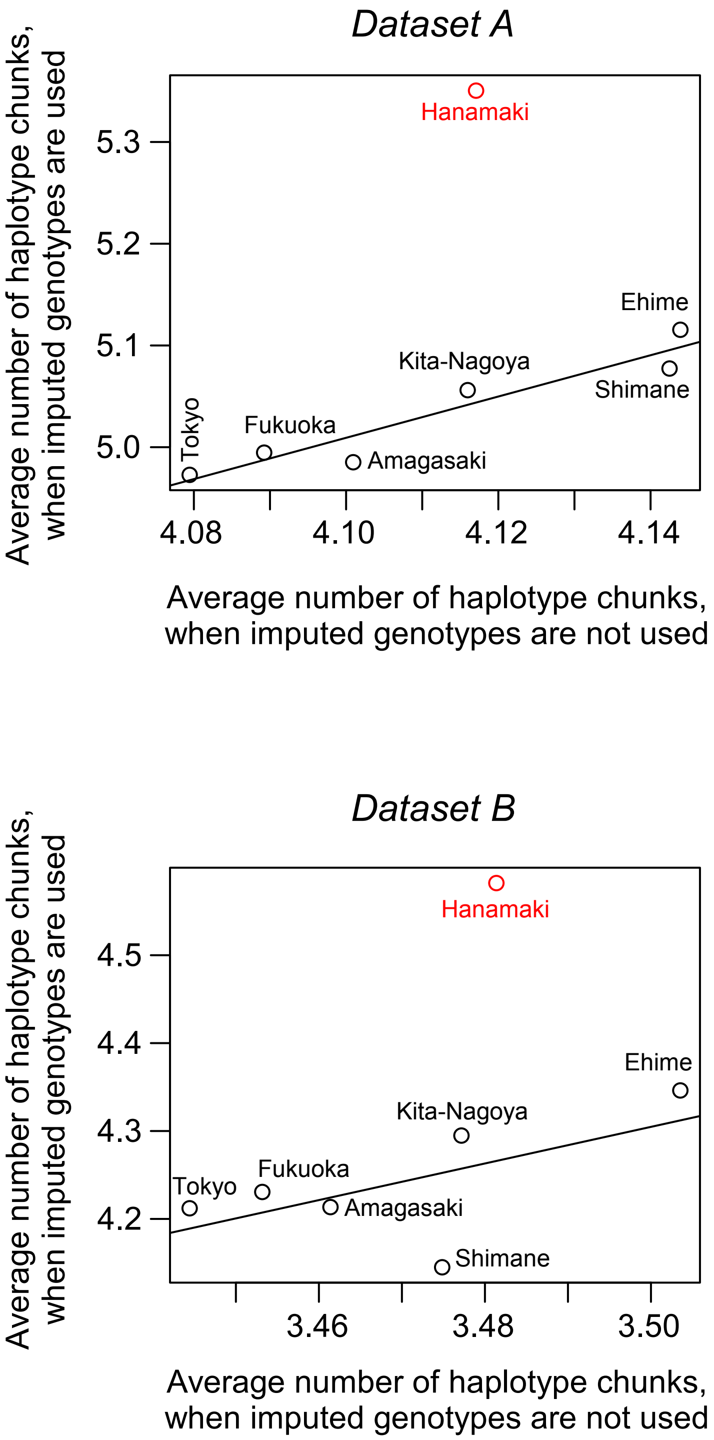

Supplement: S8 Fig — For datasets A and B, we compared the coancestry matrices computed using the imputed genotypes or not. For seven Japanese populations, the average number of haplotype chunks donated from a donor to a recipient in the same population were plotted. The Okinawa population was not included in this analysis, because it was genetically distinct from the other Japanese populations. The average number of chunks for the Hanamaki population (red circle) is slightly inflated when the imputed genotypes are used. The black line was fitted for the remaining six populations. The circle for the Hanamaki population would lie on the fitted line, if the y-coordinate is multiplied by 0.9427 or 0.9310 for datasets A and B, respectively. To the coancestry matrices based on the imputed genotypes, we applied the corresponding scaling to the sub-matrix of the Hanamaki population, and used them in the downstream analyses. The scaling canceled the inflation caused by imputation inaccuracy. (PDF) [file pone.0185487.s008.pdf]
