# Supplementary material for: The fine-scale genetic structure and evolution of the Japanese population
Source: PLoS One. 2017 Nov 1;12(11):e0185487. doi: 10.1371/journal.pone.0185487 (PMC5665431; doi:10.1371/journal.pone.0185487)

Figure S9
Dendrogram of fineSTRUCTURE clustering for *dataset A*. There are 1600 tip nodes corresponding to individuals, 1599 internal nodes, and 3198 edges in the tree. The tip nodes are aligned on the right end from top to bottom, and colored according to genetic clusters. Most of the edges near the tip nodes are very short and invisibly condensed in the right end of the figure. The tree was drawn using the FinestructureRcode that accompanies the fineSTRUCTURE program.

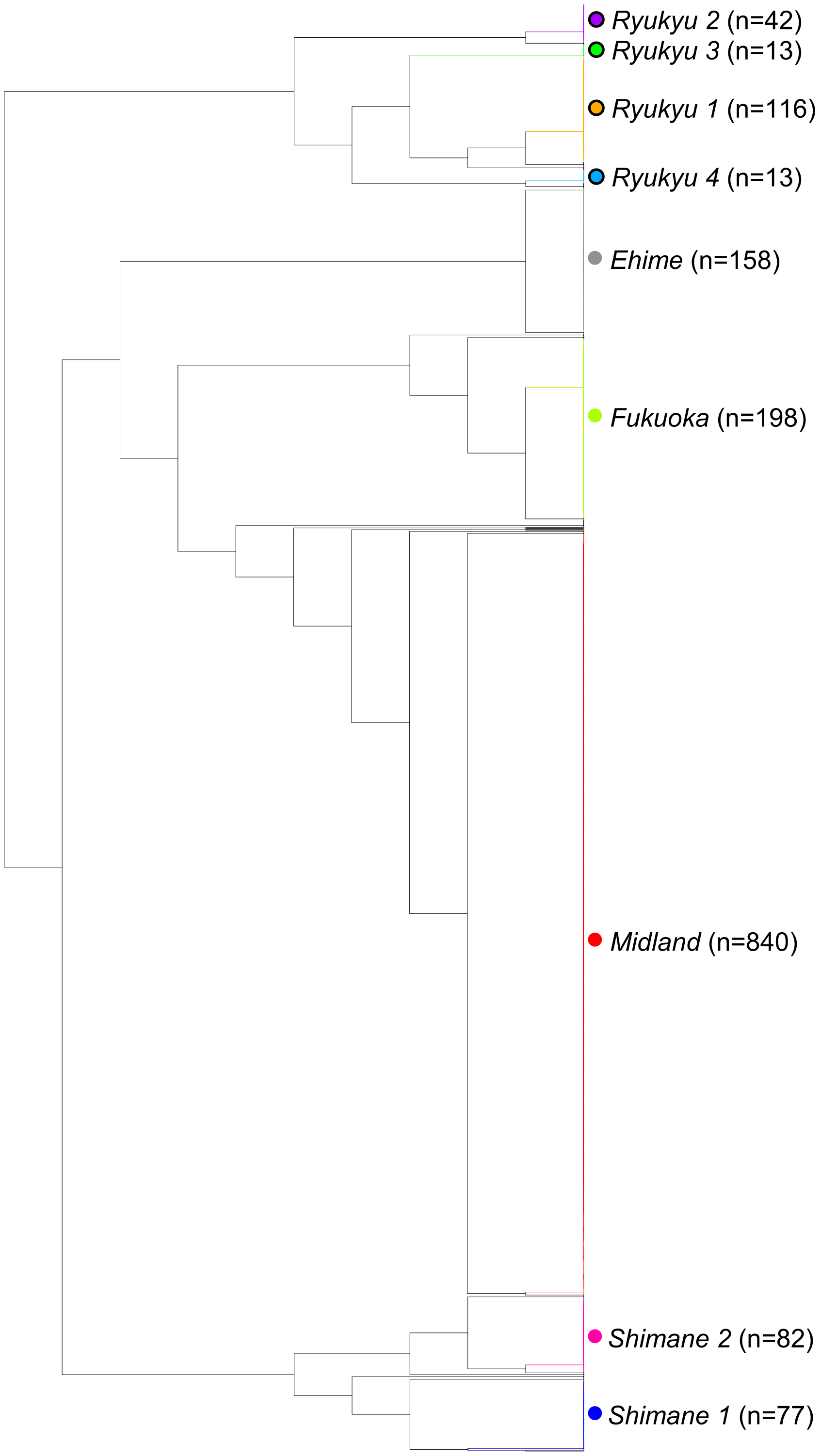

Supplement: S9 Fig — There are 1600 tip nodes corresponding to individuals, 1599 internal nodes, and 3198 edges in the tree. The tip nodes are aligned on the right end from top to bottom, and colored according to genetic clusters. Most of the edges near the tip nodes are very short and invisibly condensed in the right end of the figure. The tree was drawn using the FinestructureRcode that accompanies the fineSTRUCTURE program. (PDF) [file pone.0185487.s009.pdf]
