# Supplementary material for: The fine-scale genetic structure and evolution of the Japanese population
Source: PLoS One. 2017 Nov 1;12(11):e0185487. doi: 10.1371/journal.pone.0185487 (PMC5665431; doi:10.1371/journal.pone.0185487)

Figure S10
A pairwise coincidence matrix for two independent runs of fineSTRUCTURE clustering for *dataset A*. The (i,j) dot represents the posterior probability of the i-th and j-th individuals belonging to the same cluster. The dot is colored by the probability according to the scale shown in the right to the plot. The lower-left and upper-right parts of the plot represent two independent runs of Markov chain. Almost symmetrical patterns with the diagonal line as a symmetrical axis indicate the stability of fineSTRUCTURE clustering.

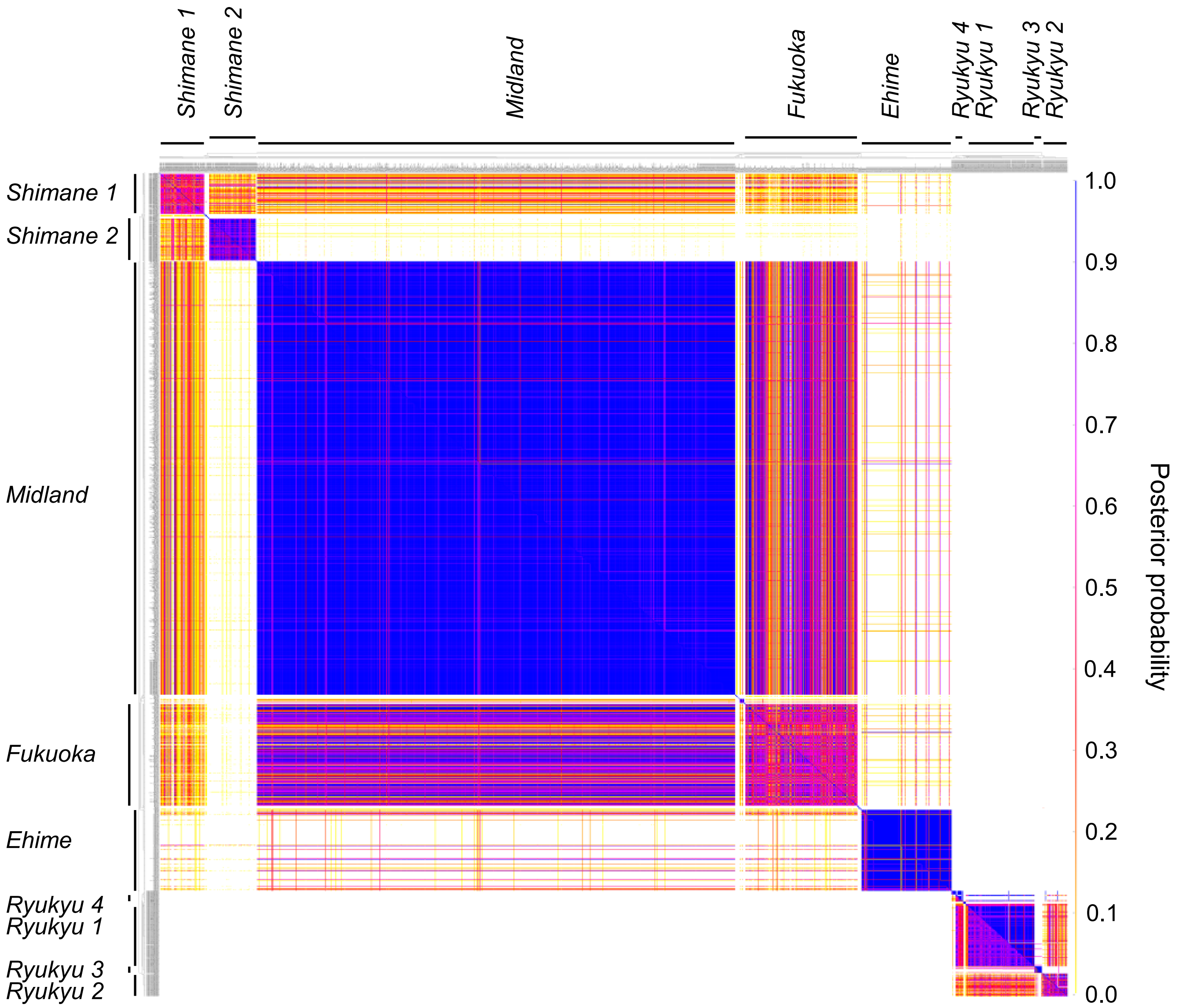

Supplement: S10 Fig — The (i, j) dot represents the posterior probability of the i-th and j-th individuals belonging to the same cluster. The dot is colored by the probability according to the scale shown in the right to the plot. The lower-left and upper-right parts of the plot represent two independent runs of Markov chain. Almost symmetrical patterns with the diagonal line as a symmetrical axis indicate the stability of fineSTRUCTURE clustering. (PDF) [file pone.0185487.s010.pdf]
